# Influences on veterinary professionals to perform wildlife medicine in the United Kingdom: A qualitative multifocal study

**DOI:** 10.1371/journal.pone.0341752

**Published:** 2026-10-27

**Authors:** Rebecca Lang, Elizabeth Mullineaux, Lian Thomas

**Affiliations:** 1 Department of Conservation Medicine, Division of the Royal (Dick) School of Veterinary Studies, University of Edinburgh, Edinburgh, United Kingdom; 2 Capital Veterinary Services, East Lothian, United Kingdom; 3 Department of One Health, Division of Global Agriculture and Food Systems, University of Edinburgh, Edinburgh, United Kingdom; 4 Department of One Health Centre in Africa (OHRECA), International Livestock Research Institute, Nairobi, Kenya; University of Zambia School of Veterinary Medicine, ZAMBIA

## Abstract

**Introduction:**

In the United Kingdom (UK) thousands of wild animals enter veterinary clinics and wildlife centres requiring medical support and rehabilitation care due to natural and anthropogenic diseases and injuries. The objectives of this empirical research were to survey veterinary professionals, wildlife centre personnel and academic educators to explore the influences of culture, working relationships, education and professional obligations on wildlife treatment experienced by the veterinary community.

**Methods:**

A mixed-method of a semi-quantitative survey and qualitative interviews was conducted. Purposive, convenience sampling was used for an online survey which was completed by 409 veterinary participants and 20 veterinary professionals, seven wildlife centre personnel and six academic educators were interviewed. The survey data was analysed descriptively and the interview data was analysed using reflexive thematic analysis.

**Results:**

Themes associated with promoting wildlife treatment composed of Competency, Motivation and Collaborations. Themes associated with inhibiting wildlife treatment consisted of Uncertainty, Lack of Resources and Conflicts. The Royal College of Veterinary Surgeons (RCVS) had an influential role on both promoter and inhibitor themes.

**Conclusion:**

A proactive wildlife medicine culture in the UK would benefit from the recognition and promotion of the values and benefits of working with wildlife. Starting with the inclusion of wildlife medicine to veterinary curricular. Support and communication within and between veterinary practices and wildlife centres to utilitise resources and information, develop networks and mitigate challenges to promote competency, motivation and collaboration. With support from the RCVS to refine educational and professional standards and enforce the regulation of the veterinary community in their provision of wildlife medicine.

## Introduction

In the United Kingdom (UK), thousands of wild animals enter veterinary clinics and wildlife centres every year. Primary causes of wildlife requiring medical and rehabilitative care include becoming orphaned*,* traumatic collisions and predation [1,2].

In 2015 over 8,000 casualties were admitted to 143 veterinary clinics nationwide [2], In 2011 over 71,000 casualties admitted to 27 rehabilitation centres [3] and between 2012–2022 over 42,800 animals were admitted to one wildlife hospital in Somerset [1]. However the combined intake of wildlife casualties at wildlife centres and veterinary clinics is difficult to determine and is potentially under-estimated due to infrequently published and widely variable data [4].

In the UK, veterinary professionals (veterinary surgeons and registered veterinary nurses) have a professional obligation to treat all animals as per the Codes of Professional Conduct [5,6], set by the RCVS, the regulating body of UK veterinary professionals. This is outlined in Article 3.7: “*The purpose of first aid and pain relief is to attend to the initial and essential welfare needs of the animal”* and Article 3.9: “*A veterinary surgeon on duty should not unreasonably refuse to facilitate the provision of first aid and pain relief for all other species”* However the treatment of wildlife is not specified nor is there any acknowledgement of the importance of wildlife for the environment and society, despite a precedent being set by other international veterinary standards like the College of Veterinarians of Ontario [7].

The current culture of the veterinary profession suggests a willingness to provide treatment to wildlife casualties. With 84% of UK veterinary professionals [2] and 94% of Australian veterinary professionals [8] administering levels of treatment beyond first aid and stabilisation, comparable to companion animals. Several limiting factors have however been identified to inhibit wildlife treatment in veterinary clinics; knowledge/skills, facilities/equipment, money/expenses, time and limited availability of competent rehabilitation centres [2,8,9]. Limited knowledge and skills has been exacerbated by a lack of wildlife medicine in college and university education [8–10].

Although the veterinary industry is an important part of wildlife treatment, their predominant responsibility is triage and first aid [11]. If euthanasia or immediate release has not occurred, the casualty will often require further support and care which is the general role of wildlife centres [12]. Therefore the veterinary and rehabilitation industries can provide critical support, advice and treatment protocols for each other [13]. However current research has demonstrated that communication issues, different opinions and conflicts over treatment can strain that relationship [8,14], leading to hostilities, antagonistic behaviours and complaints being made to the RCVS [13].

The current literature illustrates large but non-quantified numbers of sick and injured wild animals entering veterinary practices annually. That despite a willingness to treat wildlife, veterinary professionals are restricted by limited knowledge, education, resources and strained relationships between veterinary and wildlife centres. By exploring these challenges in a qualitative study from a range of voices, the aim of this research was to determine what factors currently promote and inhibit wildlife treatment. Thereby providing recommendations to improve wildlife medicine services provided by veterinary professionals.

## Methods

### Ethical statement

Ethical approval was obtained from the Human Ethical Review Committee, University of Edinburgh, Reference: HERC_2024_120.

### Survey of veterinary professionals

An online veterinary professional survey (VPS) was made available from 10 November 2024–31 March 2025. A purposive, convenience sampling approach was used for recruitment with an advert being posted on three Facebook veterinary platforms and two veterinary university online alumni platforms, after written consent was obtained from the administrator. The advert was also distributed to employees and alumni directly by the representatives of three UK-based nationwide veterinary companies and one veterinary university. Inclusion criteria were any persons working in any position in first opinion companion, equine, farm, mixed and out-of-hours veterinary practices. Exclusion criteria included persons working at referral centres, wildlife centres, laboratories, zoological institutes, tele-medicine or who were non-practicing.

Respondents were directed to a Microsoft Form survey where each respondent gave informed consent to the first information page before being able to complete the survey. The survey consisted of 20 nominal questions including yes/no and multiple-choice categories to facilitate compliance and completion with a single free-text option for additional comments. The online survey can be found at: https://doi.org/10.7488/ds/8059.

The VPS data was collected and stored on the secure Microsoft Forms online platform and downloaded for analysis in Excel. Analysis was descriptive rather than statistical to obtain basic data from the broader veterinary profession, and to compare with data from the interviewees.

### Key informant interviews

Purposive convenience sampling was used for interview recruitment, with participants sought from three groups: veterinary professionals, wildlife centre personnel and academic educators. Pilot interviews were performed beforehand. Interview recruitment and completion occurred between 10 November 2024–28 February 2025.To facilitate reaching data saturation for thematic analysis the aim was to perform at least twelve interviews per group [15,16]. A semi-structured interview was used for each group containing pre-determined core questions. Each interviewed participant completed an interview information and consent form before the interview was started. The interview scripts for each group are available (S2 Document) and can also be found at: https://doi.org/10.7488/ds/8059

Veterinary professionals were recruited via the VPS, by voluntarily submitting their email address at the end of the survey, and twenty interviews were performed. Wildlife centre personnel were recruited through contact information from the British Wildlife Rehabilitation Council website [17]. Recruitment criteria included treating “*All Wildlife*” species and showed evidence of current activity including species and numbers seen annually on their website. Email contact was made to 22 wildlife centres, of which seven participants completed the interview process. Ten academic educators at eight UK veterinary universities, that provides undergraduate veterinary and veterinary nurse courses, were contacted directly by email. Six educators completed the interview process.

The twenty veterinary professional interviews achieved the expected sample size and data saturation during the analysis with reducing novel information being obtained [15,16]. Although wildlife centre recruitment was less than expected, data saturation was achieved with less new observations during analysis. However, there was a sense of data scarcity after the university interview analysis which also didn’t reach recruitment capacity. Unfortunately, due to time restraints, recruitment was not continued.

### Interview data management and analysis

Each interview was conducted online using a laptop and was recorded and transcribed using Microsoft Teams software. Each transcript was then anonymised before deposition and manually edited for accuracy and verbatim [18]. The interviews lasted a mean average of 35 minutes and a copy of the transcript was sent to each participant. Repeat interviews were not carried out.

Reflective, inductive thematic analysis provided a flexible method of analysis for qualitative interview data [18], complimented by the survey data. This allowed a respectful exploration and reflection of participant outlooks, deductions and explanations, whilst incorporating the researcher’s interpretations [18].

The first author performed the interviews and undertook the data coding and thematic analysis. A UK-educated veterinary surgeon who has experienced wildlife casualties admitted to first opinion companion animal practice with interest. The analysis and interpretation of the data gathered should therefore be considered with her positionality in mind.

After transcript familiarisation, codes of both semantic communications and latent suggestions were manually derived from the data. Interview transcripts were then imported into NVivo software where collated codes with a central concept were consolidated into one or more primitive themes for analysis. A constructionist analysis was selected to illustrate the whole rich dataset and predominant themes.

## Results

The online survey was completed by 409 veterinary professionals. The results were summarized in Tables 1 and 2 and described in combination with the key informant interviews. The interviews were completed with twenty veterinary professionals, seven wildlife centre personnel and six university educators. Codes, subthemes and themes were developed using thematic analysis (S1 Table). A conceptual framework (Fig 1) illustrates three themes that promote engagement with wildlife treatment: Competence, Motivation and Collaborations. Three themes that inhibits engagement with wildlife: Uncertainty, Lack of resources and Conflicts, and a central influence by the RCVS. The The anonymized survey results and interview transcripts can be found at: https://doi.org/10.7488/ds/8059.

**Table 1:** Veterinary professionals survey demographics.

Topic	Options	Results (n = 409)	Percentage
**Professional Role**	Veterinary Surgeon	235	57%
	Veterinary Nurse	110	27%
	Owner/Director/Manager	52	13%
	Animal Care Assistant	5	1%
	Student	4	1%
	Receptionist	3	1%
**Age**	20-29	99	24%
	30-39	147	36%
	40-49	101	25%
	50-59	47	11%
	60	15	4%
**Veterinary Practice Type**	Small Animal	291	71%
	Mixed	64	16%
	Dedicated OOH Provider	34	8%
	Exotic	8	2%
	Equine	5	1%
	Farm animal	4	1%
	Other	3	1%
**Business Type**	Corporation	237	58%
	Independent single branch	73	18%
	Independent multiple branches	68	17%
	Charity/Non-profit	20	5%
	Locum	7	2%
	University	4	1%
**Education Received**	UK Veterinary University	250	61%
	Didn’t attend university	63	15%
	Overseas veterinary university	49	12%
	College/apprenticeship	27	7%
	Other UK university	20	5%

**Table 2:** Veterinary professionals survey results.

Topic	Options	Results (n = 409)	Percentage
**Wildlife Medicine in Undergraduate education**	No wildlife medicine taught	130	32%
	Occasionally added to other topics	118	29%
	I can’t remember	69	17%
	Core subject	33	8%
	Extra-curriculum lectures or clubs	27	7%
	Elective subject	22	5%
	Not applicable	10	2%
**Interest in wildlife medicine continual professional development (CPD) or training**	Yes I have undertaken wildlife CPD/training	117	29%
	Yes and I would like to do more	90	22%
	No but I would like to undertaken some	132	32%
	No and I’m not interested in wildlife training	67	16%
**Interest in wildlife volunteering**	No Interest	253	62%
	Yes Currently volunteer	66	16%
	No but I’d like to volunteer	90	22%
**Resources used to treat wildlife (multiple choice)**	Wildlife Rehabilitation centres	279	68%
	Colleagues/Peers	276	67%
	Internet search engine	256	63%
	Textbook on wildlife species	223	55%
	Textbook on exotic species	193	47%
	Own lecture notes or CPD notes	212	52%
	Other	18	4%
**Relationship with Wildlife Centres**	Good relationship with one or more wildlife centres	174	43%
	Occasional contact with a local centre	146	36%
	Relationship present but could be better	55	13%
	No communication with any centres	34	8%
**Practice Protocols and SOPs**	We have some vague protocols or SOPs	130	32%
	We don’t have any wildlife protocols or SOPs	124	30%
	We have detailed wildlife protocols and/or SOPs	72	18%
	I don’t know if we have any	70	17%
	We have some but I have not read them	13	3%
**Communication with the Public**	No but I think we should;	147	36%
	No and I don’t think we should	142	35%
	Yes, website/socials/notice boards and/or collect donations/raise funds for wildlife charities	117	29%
**Awareness of RCVS investigation** **of wildlife complaints**	Yes	169	41%
	No	241	59%

**Fig 1:**
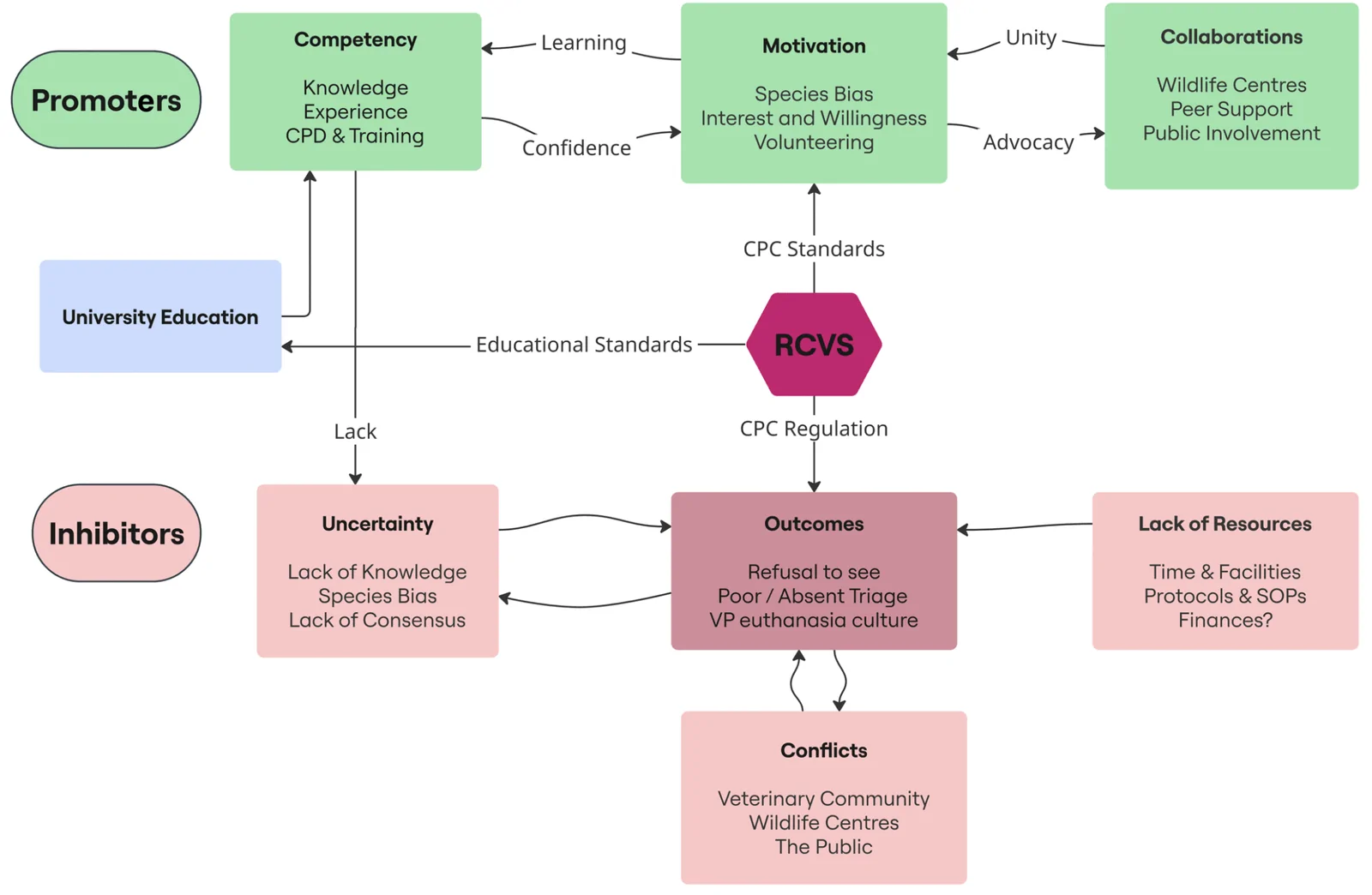
Conceptual framework of themes and subthemes.

The demographics of the surveyed veterinary professionals (n = 409) included professional role, age, veterinary clinic type, business type and education received (Table 1). In summary, 57% of participants were veterinary surgeons and 27% were veterinary nurses. Over a third (36%) were aged between 30–39 years followed by 25% aged between 40–49 years. The majority (71%) worked in small animal practice, 58% worked for corporation practices and 61% attended a veterinary university in the UK.

### Promoter Themes

Promoters that encouraged and enabled wildlife treatment by veterinary professionals consisted of Competency, Motivation and Collaborations.

#### Competency.

Knowledge and experience were discussed by exploring the sources of learning, undergraduate education and continuing learning, and their benefits.

Regarding education, most veterinary interviewees conveyed that, from their experience, UK college and university curriculums were lacking quality wildlife medicine. Either wildlife medicine was present but combined with exotic medicine, was part of an elective, only available through self-organized extra-mural studies or didn’t feature at all, although there was the possibility of poor recall. Reduced student awareness or appreciation of wildlife teaching was also discussed, resulting from potential ignorance of wildlife cases being seen in a veterinary setting.

*I was at university from 2012 to 2017 and I can’t remember anything wildlife specific (VP03). We got some exotic lectures and then that’s what you just apply to wildlife (VP10). [Wildlife] certainly wasn’t something that massively appealed to me as an undergraduate… I don’t think maybe as undergraduates you appreciate how much wildlife you might see and how useful it would be to have those tools (VP08).*

Nearly half the VPS (49%) stated they either didn’t receive or couldn’t remember any wildlife medicine during their undergraduate education with only 8% stating wildlife medicine was a core subject in the curriculum (Table 2). However, a trend towards an increase in wildlife medicine education over time was identified, particularly after 2013 (Fig 2), although a level of recall bias should be considered.

**Fig 2:**
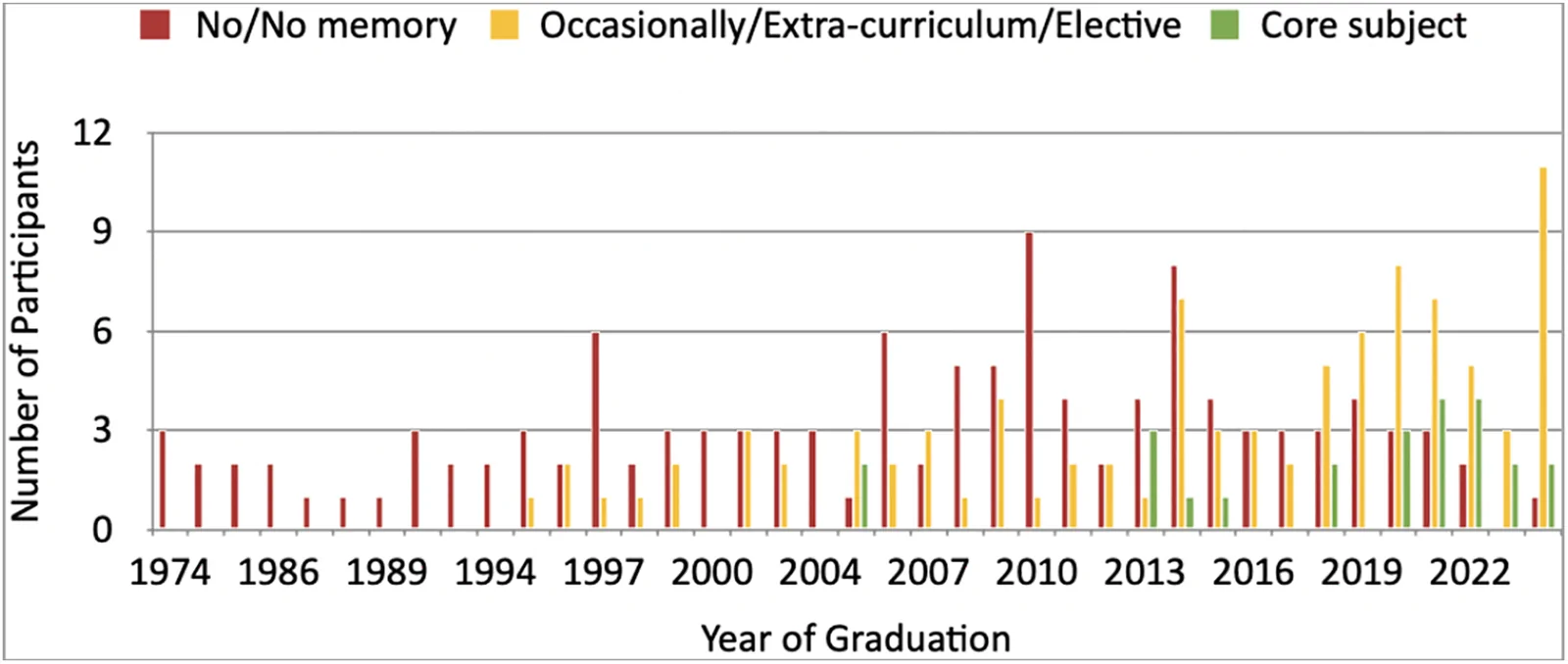
Inclusion of wildlife medicine in university curricular over time.

Interviews with academic educators gave the opportunity to discuss the inclusion of wildlife medicine into their curricular. For some, wildlife medicine had been included as a core or elective subject for several years, for others the subject was included with exotic medicine with the expectation that students applied the basic principles taught to wildlife species. For one educator wildlife was absent from the curriculum. All educators agreed that wildlife medicine was a relevant and valuable topic for students and were happy to discuss how wildlife education could be incorporated more into future curricular.

*There is definitely [wildlife] training in the core program. … it would have been 2014 that that module ran for the first time (AE03). We feel it is important to include wildlife teaching in our undergraduate curriculum, however many of the avian, reptilian and mammalian practical skills we teach in our exotics course are directly extrapolated to UK wildlife species (AE02). Currently we don*’*t have anything wildlife medicine or rehabilitation at all on our curriculum which in one way I was quite surprised to discover (AE05).*

After graduation, the variety of further learning and development opportunities experienced by veterinary professionals included volunteering at wildlife hospitals, conference lectures, in-house or external training courses, online webinars, advanced qualifications and in-practice exposure. With the main benefits of increased knowledge and experience leading to improved confidence and competency.

*We’ve been doing some more in-house training on birds, particularly the seabirds (VP10). I went to British Veterinary Association live in the summer and I went to a couple of their specific wildlife lectures (VP18).* We tend to get a lot of pigeons…I’m pretty conﬁdent with handling them and giving them a full kind of examination *(VP07).*

In the VPS, there was a high level of interest in gaining and improving wildlife medicine knowledge with 83% of participants having either already, or would like to, undertake further wildlife training (Table 2).

#### Motivation.

All the interviewed veterinary professionals expressed a willingness and interest in working with wildlife, so several intrinsic motivations were described. Wildlife had either been a life-long passion or an aspect that developed during their veterinary career. Motives including the enjoyment of a challenge; helping animals being a fundamental principle; personal fulfilment from providing help and aid; or concerns about wildlife welfare.

*It’s when you feel like you’re being a veterinarian and you’re really helping things and it’s interesting you know because it’s not something you do absolutely every day (VP13). It’s still absolutely brilliant watching their little [hedgehog] butts waddle away as you get to release them (VP09).*

In the VPS, extrinsic motivators to treat wildlife were ranked higher. The top-ranking influence was Welfare and the Reduction of Suffering, with Passion and Interest in Wildlife ranking last. (Fig 3)

**Fig 3:**
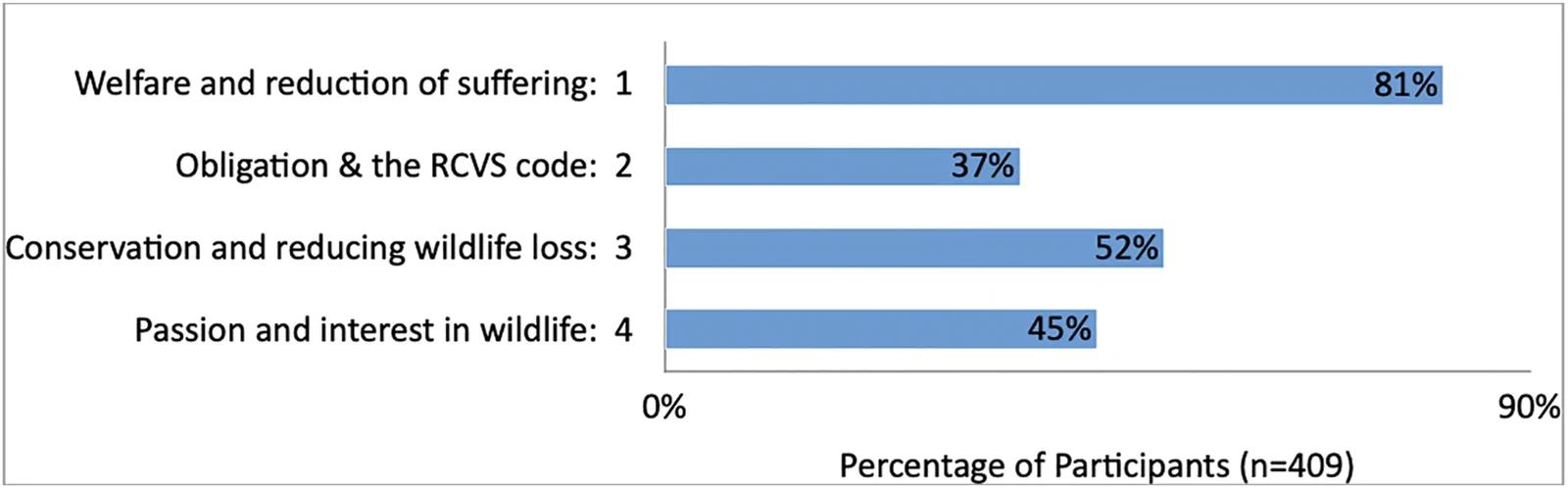
Survey participants’ ranking of influences to treat wildlife.

The act of volunteering with wildlife outside of the veterinary workplace demonstrated further intrinsic motivation. Various activities described included memberships or volunteering with wildlife organisations, transporting wildlife to rescue centres, offering wildlife training, producing online content and providing rehabilitation opportunities in the home. In contrast 62% of the VPS expressed no interest in volunteer work with wildlife.

*My social media page that I run… I’m still very much like an advocate for wildlife (VP07). I do a hedgehog rescue as well at home (VP19). I don’t mind transporting them there, but it is a two hour to three hour round trip, whichever rescue we choose (VP03).*

The willingness to treat certain species or classification of wildlife was evident. The main reasons for species bias were increased exposure leading to more experience and increased confidence with that species. Although an appreciation for certain rarer or unique species was another factor. Hedgehogs were the most commonly treated and appreciated species in the interviews, a bias observed by both veterinary and wildlife personnel. In the VPS, hedgehogs also received the highest vote (30%) for favourite species (Fig 4).

**Fig 4:**
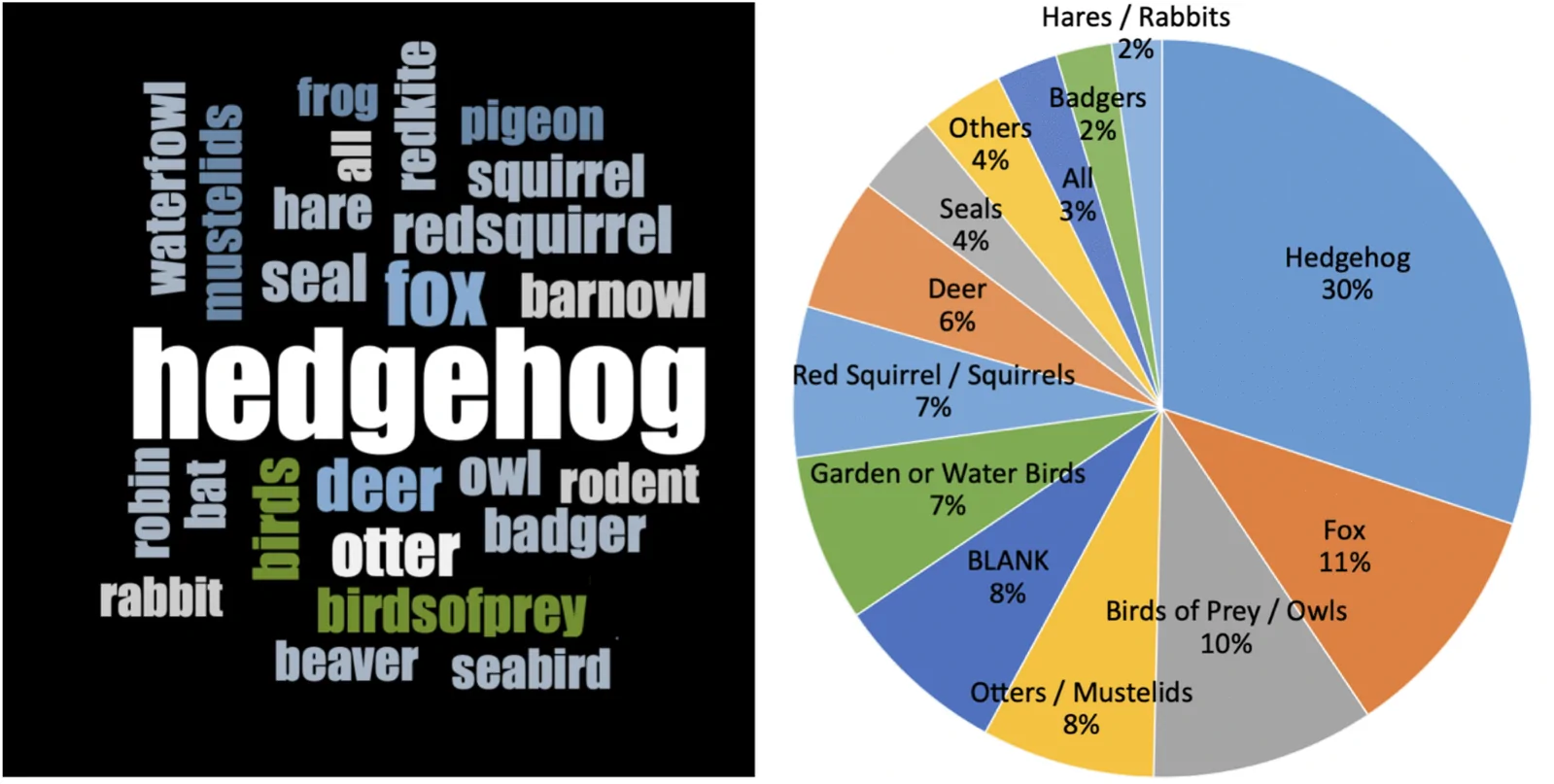
Word cloud of positive species bias from the survey.

*I’m really confident, especially with the hedgehogs, because we do a lot of hedgehogs (VP10). Veterinarians are much more willing to look at and treat hedgehogs. They’re much more willing to like, properly assess them. They’re more comfortable with them (WC02). On the Isle of Wight we have red squirrels … and everyone tries to do what we can to save them (VP17).*

#### Collaborations and communication.

Collaborations identified during the interviews included within the veterinary community and with wildlife centres and the public. Examples of peer support within and between the veterinary community included sharing wildlife resources and knowledge gained from experience or training and developing a network of contacts for enquiries and advice. In the VPS, the most commonly consulted wildlife resources were wildlife rehabilitation centres and colleagues and peers, (Table 2).

*I have tried to like create a bit more of a protocol and an examination checklist. So that literally any one of the members of the team can at least go through like the initial stages (VP07). I’ve got a good support network that I can just message and go “look at this X-ray and what do you think?” (VP12). Some of the local practices I gave them my number purposefully to say* “*look if you ever get a hedgehog don*’*t be afraid just ring me” (VP19).*

Encouraging working environments and attitudes towards wildlife within the practice were discussed by several veterinary professionals, either initiated by themselves or colleagues leading by example, including supportive management and legacies established by previous colleagues or management.

*The moment I suggested [wildlife CPD] was something I wanted to do, the practice jumped on it and said “yeah, we want somebody to be a keystone for this and then filter the knowledge through the rest of the team” (VP09). I have got the practice to see a bit of [seals] because of my own personal interest (VP18). One of our bosses… his premise is always strays or whatever get treated, so you know we’ve always done a lot (VP10)*

Collaborations also extend to others who work with wildlife, predominantly wildlife rehabilitation centres and hospitals. The VPS showed 42% of participants selected a good relationship with wildlife centres and 49% stated they had occasional or “could be better” relationships. (Table 2) The reasons for veterinary professionals communicating with wildlife centres included asking about species identification, biological or ecological considerations, treatment or prognosis guidance and transfer information. The peace of mind and feeling of support this relationship generated was also described.

*So we would, mostly go to [wildlife centre] if we want to discuss things, you know, for advice, if they think that this is something they can rehabilitate (VP02). It’s not my area of expertise and having that phone a friend support … to someone who does have the expertise and the knowledge. It is really important for me (VP08).*

From the interviews with wildlife rehabilitators, the collaborations instigated by wildlife centres with veterinary practices included; utilisation of veterinary clinics for medical, surgical and euthanasia cases and the opportunity for mutual advice, support and training. Empathy was also expressed for the veterinary community regarding the foreignness of wildlife treatment.

*The veterinarians that work really well with us are the veterinarians that have actually come into the centre and met us and done our CPD and that kind of thing (WC02). I have all the sympathy in the world for a veterinary surgeon working a small animal practice being presented with a casualty they’ve never handled before and all the expectation of the world for them to fix it (WC04).*

The public was another sector discussed by both veterinary and wildlife interviewees in relation to collaboration and communication. The benefits of veterinary clinics communicating their wildlife work to the public were described as improved public relations, reduced negative perception towards the veterinary profession, a source of education, reduction of erroneous casualties and even a boost to business. However, some interviewees were concerned that advertising wildlife work would have a snowballing effect on caseloads. The VPS demonstrated a split opinion with 29% actively communicated with the public about wildlife, 36% thought they should and 35% % thought they shouldn’t. (Table 2)

*I think it’s important for the public to know that you treat wildlife, because we get enough stick as it is for just being in it for the money* (VP10). *You take one hedgehog, you share its story on social media. It’s seen by 30,000 people* (WC04). *A lot of people started using his practice because of his association with the wildlife rescue* (WC01). *If you then become the clinic that’s known to see wildlife and deal with wildlife, then I think that then becomes a self perpetuating story and that you will then see more wildlife because people will be aware that (VP06).*

### Inhibitor themes

Inhibitors that challenged and restrained veterinary professionals from treating wildlife casualties consisted of Uncertainty, Lack of Resources and Conflicts.

#### Uncertainty.

A feeling of uncertainty arose from veterinary interviewees when discussing limited knowledge, a lack of treatment consensus and scant experience with certain species. These were represented by a deficiency in experience and confidence, fear of the unknown, frustration and indecision regarding differing opinions on appropriate treatment options, lack of clarity and direction from management and layperson unawareness of professional obligations toward wildlife.

*[Wildlife] is often something that you’ve never treated or potentially never handled before and I think it’s just quite a daunting thing (VP18). I see in lots of the social media groups and things discussions all the time about should I amputate this hedgehog’s leg? You know, should I do this? (VP06). Sometimes when you hear the veterinary practice refused to see [wildlife], it was actually the receptionist who refused to see it and the veterinarians not heard anything about it (WC04).*

It was evident that limited knowledge of ecology, medicine or handling of a species can result in a bias against that species. The VPS demonstrated a species bias against; Rats (14%), Pigeons (10%) and Badgers (8%) (Fig 5). Uncertainty can then compound other factors of bias including health and safety, volume overload, phobias or vermin classification.

**Fig 5:**
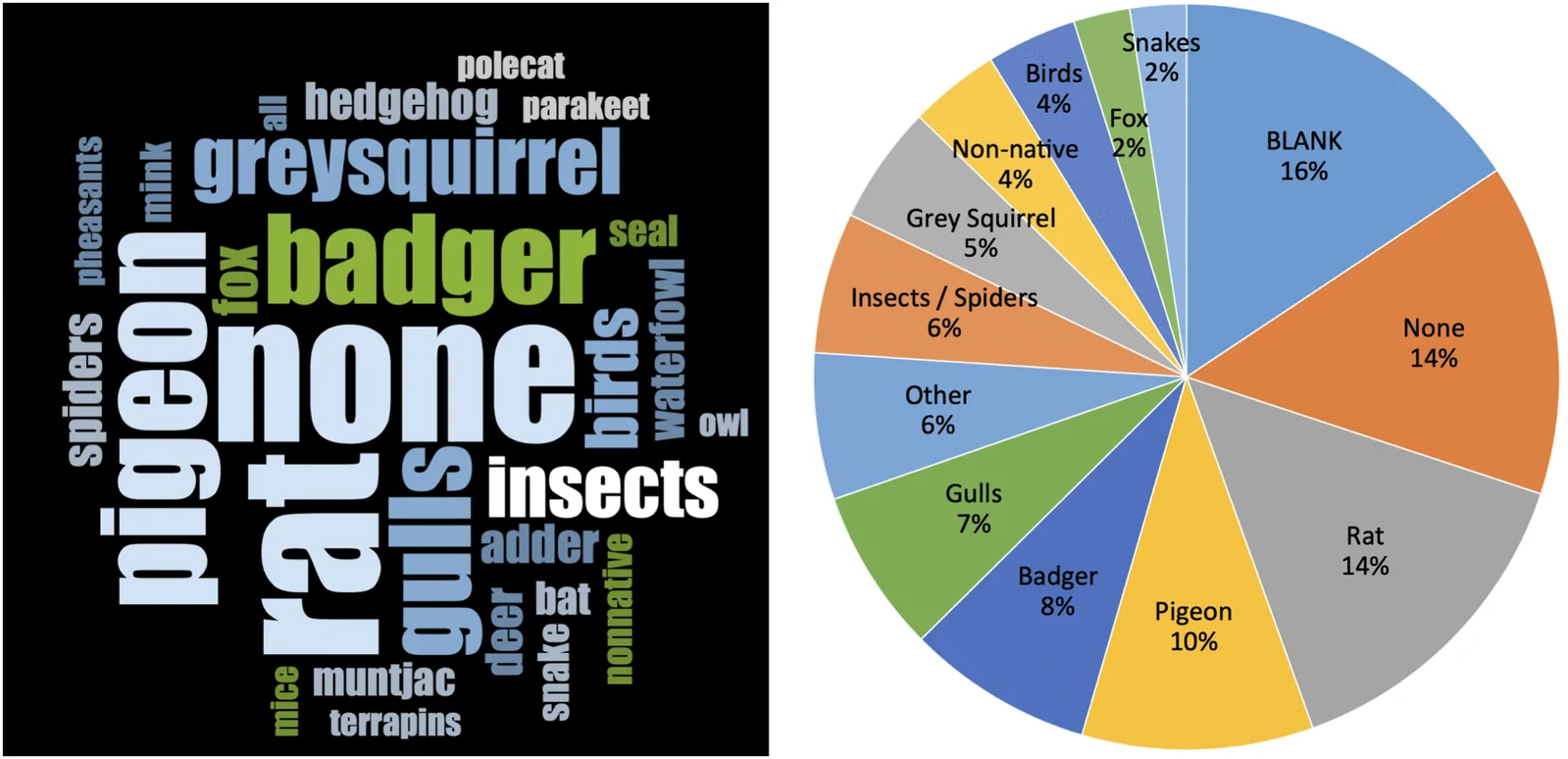
Word cloud of negative species bias from the survey.

*If I felt I had a little bit more knowledge and capability I could maybe do a bit more, especially with the birds (VP01). Obviously with bats I know that you have to be really careful with you know rabies and things like that (VP05). We just get so inundated with birds and fledglings. I have a really negative reaction to it… especially pigeons… the winged rats of the world (VP17).*

These facets of uncertainty; lack of knowledge, experience, confidence and a bias against a specific species or wildlife in generally were all described as features for euthanasia decisions made directly or witnessed by the interviewees.

*I think sometimes when you feel that lack of confidence around the decision making, then euthanasia decision seems like a safe one in terms of welfare (AE03). The new head veterinarian is somebody that doesn’t really treat wildlife. She would just euthanise anything that came in (VP16).*

#### Lack of resources.

The lack of resources that limited wildlife treatment and exacerbated euthanasia decisions were identified as insufficient time, facilities and protocols. Busy practices, stretched personnel, limited hospitalisation facilities and a lack of transfer options to wildlife centres were discussed as inhibiting factors to treat wildlife. The consequences of which were described as demoralisation, frustration and an increase in euthanasia decisions.

*We don’t always have enough time to provide as proficient care [to wildlife] as we would for others (VP17). It’s not appropriate to keep [wildlife] with dogs barking and cats in the environment with the prey animals (VP03). It’s a little bit difficult and that puts ethical constraints on to it as well because if there’s nowhere for them to go and they’re not fit to be released in the wild, they often end up only being one option! [euthanasia] (VP18).*

A lack of finances and funding was mentioned by most interviewees as a possible or potential inhibitor, however it was then often followed by a comment suggesting it wasn’t a true inhibitor for them.

*I think sometimes funds are a bit of an issue … but it definitely wouldn*’*t stop me from you know doing my part (VP05). It’s not costing us very much, to provide a little bit of comfort to these animals. They’re not doing any harm sat in a kennel for a few hours whilst we work out a plan with a little bit of pain relief on board (VP18).*

When asked about using wildlife protocols and standard operating procedures (SOPs), interviewees responded that either there were none in the practice, some protocols were in a cupboard or draw somewhere or despite easy access they were not being used by other members of staff, for example admission forms for reception staff. The VPS showed only 18% of respondents had detailed wildlife protocols and SOPs.

*Any veterinarian in the building that’s available who will examine it and make a decision of what’s happening. And it’s just very much individual. There are no protocols whatsoever (VP18). We’ve done [wildlife] forms but that doesn’t mean they’re gonna use them (VP12).*

#### Conflicts.

Conflicts about wildlife were found to have inhibiting connotations within a team, between veterinary and wildlife organisations and with the public.

Conflicts experienced or perceived by interviewees within the veterinary practice and wider community ranged from differences in opinion of treatment options, species bias, lack of interest, reluctancy or even refusal to see wildlife. This produced frustration and conflicts when peers were not working within the veterinary Code of Conduct and not taking their share of the wildlife caseload.

*One of [the veterinarians] is very against treating pigeons because she sees them as vermin (VP10). Nurses actually threatened to leave the practice because I’ve insisted we euthanise [grey squirrels] because it is illegal to release one once it’s trapped (VP15). The amount of people that I see in my local area that contact veterinarians with wildlife needing first aid that are turned away is quite high, which I find really disappointing (VP16).*

Tension over wildlife is not confined within the veterinary industry. The veterinary interviewees also described multiple conflicts they experienced with wildlife centres. These included a lack or vague responses to queries about advice, training, case feedback, establishing a relationship, transfer options, accepted species and also concerns about moral and ethical standards at some backyard rehabilitation locations.

*I have reached out to a couple of bigger wildlife hospitals and actually not heard anything and that’s been a bit frustrating (VP13). They never answer their phone so that makes it quite difficult for us if we’ve got cases that are potentially slightly borderline as to whether or not they can be rehabilitated, or cases that we’re not sure whether they would necessarily accept (VP18). There are a lot of folk around here with multiple rabbit hutches full of seagulls which I don’t think is appropriate (VP15).*

From the viewpoint of the wildlife centre interviewees, their main concerns were veterinary clinics refusing to see wildlife, convenience euthanasia and prolonged and variable triage with a vague understanding of treatment options.

*It’s not just treated incorrectly or euthanised on the spot for being a wildlife casualty. It’s far worse than that… where it’s a flat-out refusal. It’s a “please leave this practice, we do not do wildlife” (WC04). We get an awful lot of birds of prey come in that have like quite glaringly, obvious injuries that would mean that they need to be euthanised, that have been missed (WC02).*

Another area of conflict expounded on by both veterinary professionals and wildlife centre personnel was with the public. Requests experienced by the interviewees included locating wildlife that had run or flown away, extreme and expensive procedures, wanting to perform home rehabilitation instead of accepting euthanasia, attempting home care and then expecting a miracle save after the animal had deteriorated or procedures resulting in lifelong captivity. Several veterinary and wildlife centre interviewees had experienced, or were anxious about, aggressive behaviour and social media attacks if euthanasia was discussed or performed and that there was a public assumption that veterinarians only euthanise wildlife.

*They [want to] take it home and they’ll try and do it themselves… its never going to fly again… that, to me, is cruel (WC04). If the finder of the wild animal is not on board with that [euthanasia] explanation, then you might face a bit of a social media backlash (VP03). There are some Facebook groups saying, “don’t take it to this practice. They’re just going to kill everything” (VP17).*

### Regulatory framework

During the interviews the three main responsibilities of the Royal College of Veterinary Surgeons (RCVS) were discussed; establishing the educational standards, setting the Codes of Professional Conduct (CPC) for the veterinary profession and regulating the compliance of veterinary professionals.

When discussing of the CPCs concerning wildlife, two main responses were produced. Either that the Codes were adequate, and that only required a general guideline was necessary or that the Codes was ambiguous when it came to wildlife. The VPS illustrated that 59% of participants were unaware about the RCVS investigating complaints involving wildlife (Table 2).

*I do think to a certain extent that [the code] is sufficient… I wouldn*’*t like to take too much decision making away from the veterinarians (VP09). [The code] is open to interpretation and some people will do a bit more and others won*’*t* (VP10).

When it comes to setting the education standards, the academic educators agreed unanimously that wildlife was not specified in any of the educational standards or day one competence appendices.

*It*’*s not currently listed on the day one competences or day one skills list. It just says a broad range of species (AE05). In the appendices, core subjects to be included in the syllabus…wildlife is not mentioned at all… So yeah that*’*s it in the standards for Royal College (AE01).*

The regulation and investigation of complaints and misconducts involving wildlife by the RCVS yielded generally contemptuous responses during the interviews. This included unfit for purpose, wildlife care being of low priority and an impression of limited regulation.

*They [RCVS] probably have bigger fish to fry in their opinions than the wildlife side of things. You know, it*’*s probably fairly low down their agenda of priorities (VP08).* They *[a veterinary clinic who refused to see a wild animal] did get reported to the Royal College who brushed it off and said, well, you know, you don*’*t have to treat species that you*’*re unfamiliar with (VP15).*

## Discussion

This study has performed a deep dive into the culture and attitudes of key actors who influence how wildlife casualties receive medical treatment in the UK. In this discussion we combine the rich qualitative interview data with the survey data and place it within the context of relevant literature.

The themes identified, which promote or inhibit wildlife treatment, demonstrated reinforcing causal relationships (Fig 1). For promoters; improved knowledge, experience and training leads to more confidence, competency and positive species bias. This can produce intrinsic motivations demonstrated by personal interest and additional volunteering, increasing exposure and advocacy for wildlife, leading to collaborations and networking with like-minded others giving a sense of unity in the face of a common cause. All of which feeds back to increase motivation which continues to escalate exposure and thus competency.

For inhibitors; uncertainty results from limited knowledge, poor treatment consensus and species persecution. This, in combination with a lack of resources especially protocols, plus conflicts within and between organisations and the public, exacerbate negative outcomes of limited or inappropriate triage and treatment, a reluctancy or refusal by veterinary practices to see wildlife cases and a culture of convenience wildlife euthanasia. These outcomes feedback into deepening conflicts, demoralisation and reduced wildlife interactions, further limiting the opportunity to acquire knowledge and experience leading to more uncertainty and a-motivation.

### Competency versus Uncertainty

This study indicates that currently the education of veterinary surgeons and nurses in the UK is lacking quality wildlife medicine training. This was a recurrent topic raised by most interviewees including some educators, and almost half of the survey participants. This is surprising considering that most veterinary surgeons and nurses entering practice will engage with wildlife casualties at some point with increasing demand [10,19] and education is fundamental to appropriate animal care and welfare [10].

Both veterinary and educator interviewees described how minimal wildlife medicine in the curriculum can shield students from the realities of encountering wildlife during their clinical careers. Interviewees also discussed that limited knowledge, poor treatment consensus and a lack of exposure with certain species, especially in combination with health and safety concerns, led to uncertainty, anxiety and a potential bias against that species, which could result in inadequate care and treatment [4,19]. Barnes, Haering et al. and Varu and Orpet all found their respondents voted a ‘lack of knowledge’ as a primary inhibitor to treating wildlife [2,8,9]. Barnes also reported participants expressing concerns about quality of care, welfare and low success rates as a result of limited knowledge, skills and experience [2]. Whilst Haering et al. reported that 22.9% of respondents considered lack of knowledge as a factor in euthanasia decisions [8]. However, Mullineaux and Pawson point out that inadequate wildlife knowledge can lead to delayed euthanasia and increased welfare issues due to the failed recognition of casualties with poor prognosis or long captivity requirements [1]. There is therefore a balance between identifying poor prognosis or welfare cases for euthanasia without prolonging or hurrying an euthanasia decision due to uncertainty.

Our data provides evidence of current efforts to mitigate this knowledge deficit. The VPS survey already suggests an increase in wildlife medicine entering the core curricular at some veterinary universities, particularly after 2013. For example the University of Liverpool and Stapeley Grange Wildlife Centre in Cheshire [20] developed the first wildlife rotation for final year students in 2013 [21]. Ensuring that students are exposed to wildlife as an intrinsic component of their studies rather than assuming they will be exposed during extramural studies. However, the recognition and appreciation of wildlife medicine between the veterinary schools remains inconsistent [11] which was clear from the range of responses from the academic educator interviewees, and therefore veterinary educational standards should be reevaluated.

Even with a lack of education, many veterinary professionals still develop an interest in wildlife medicine and are keen to learn more. Acknowledging the inherent bias due to the recruitment strategy, we found that the majority of survey participants had engaged with or wished to engage with wildlife training and learning. These results were consistent with Varu and Orpet who found that although 76.7% of their veterinary nurse participants received no wildlife education, 74.2% were interested in further wildlife training [9]. Panto also found a high demand for wildlife training by veterinary professionals, which led to increased confidence, interest and an improved approach towards wildlife casualties [21]. There are increasing wildlife specific resources available to veterinary professionals including; the online, multi-collaborative Wildlife Toolkit [22], books like the Manual of Wildlife Casualties [23] in its second edition and wildlife specific conferences [24]. All aiming to provide information, facilitate treatment consensus and offer solidarity to veterinary professionals.

The positive feedback loop between affinity (as indicated by participants choice of ‘’favourite” species), knowledge, experience and confidence with certain species like hedgehogs, was described at length by the interviewees. Barnes and Varu and Orpet demonstrated a significant correlation between total knowledge and competency and the quantity of wildlife treated [2,9]. Varu and Orpet also correlated competency with most presented species to veterinary clinics (hedgehogs, garden birds and rabbits) with received education of domestic birds and rabbits [9]. In the survey, hedgehogs were voted most popular in conjunction with the interviewees, and a negative bias was illustrated against rats, pigeons, badgers and gulls by both survey participants and interviewees. Although the reasons for the survey species biases were not explored, the interviewees offered some explanations, for example, health and safety and the classification of vermin. Species bias is a reality and could be used as a starting point to promote wildlife treatment within a clinic, with autonomy to treat favourite species and exploring the reasons behind the prejudices towards other species.

### Motivation versus Resources

Intrinsic motivation is when the incentive to perform a task is the enjoyment of the task itself [25]. The motivation and benefits of working with wildlife discussed during the interviews, and mirrored by Barnes’ interviewees [2], included the enjoyment of the challenge, satisfaction in increased abilities, personal fulfilment, team achievement and experiencing something novel. The survey data however produced different orientations of motivation. The primary motivator was welfare and the reduction of suffering, follow by obligations and the RCVS code then conservation and reducing wildlife loss (Fig 3). These motivators are extrinsic, driven by anxiety avoidance or external control [25]. The lowest survey motivator was the intrinsic option of passion and interest in wildlife and this is reflected in only a third of survey respondents stating they were interested in volunteering with wildlife. This suggests that currently, despite the bias of the veterinary interviewees, the surveyed veterinary community work with wildlife due to extrinsic motivations of external pressure rather than an intrinsic internal satisfaction.

This lack of interest and intrinsic motivation by the veterinary community could in part be caused by a lack of resources, in addition to a lack of knowledge as previously discussed. Limited resources identified by the interviewees were limited time in busy practices and poor facilities hospitalising wildlife near noisy predator species. Barnes and Haering et al. also affirmed facilities/equipment and time as primary limiting factors to treat wildlife in a veterinary setting [2,8]. Whereas exotic practices had the highest competency score due to better facilities, equipment and aptitude, according to Varu and Orpet [9], demonstrating a correlation between appropriate facilities and wildlife caseload.

The question of who pays for wildlife treatment is a seesaw of opinions. Our veterinary interviewees mentioned finances as a potential inhibitor but then often followed up with a comment suggesting it wasn’t a true inhibitor for them personally. A certain level of autonomy appeared to be gifted to them by management allowing for the driving motivation to treat wildlife to be expressed and maintained [25]. Despite finances not being a question on the survey, three survey participants commented that the veterinary industry should not be expected to fund wildlife treatments whereas two others added that finances should not inhibit appropriate treatment. Both Barnes and Haering et al. reported finances being inhibitors of wildlife treatment. Barnes found 22.7% of veterinary practices did not believe the veterinary profession should invest time and funds into wildlife treatment [2], and Haering et al. found 24.8% of participates cited treatment costs as a euthanasia consideration [8]. Indeed if appropriate costs for treatments are not obtainable then euthanasia is preferable over poor care [12]. However Varu and Orpet found that charity practices would see more wildlife species as they were more accustomed to providing care with limited financial options [9] illustrating that effective care doesn’t have to be expensive.

Another resource, under-utilised by the veterinary industry, despite proficient use in wildlife centres, were wildlife protocols and SOPs. The majority of interviewees and survey participants (82%) acknowledged either vague, absent or neglected wildlife protocols in their practices. This in turn can exacerbate uncertainty when wildlife is presented to the clinic with assessment and treatment planning can take more time and cognition rather than having an initial simple standardized protocol to follow. The lack of wildlife protocols and SOPs in veterinary clinics was mirrored by Barnes, who found only 24.6% were regularly using wildlife SOPs and concluded that better use of SOPS could improve wildlife workload [2]. The interviewees agreed that limited resources of knowledge, time, facilities and finances all exacerbated a euthanasia outcome. This caused demoralisation and anxiety and could further reduce intrinsic motivation to work with wildlife. Therefore wildlife protocols for common species and injuries are an efficient resource that can; promote prompt effective triage and first aid; reduce cognitive fatigue and research time; reduce stressful delays to both animal and clinician and prevent premature transfer to wildlife centres if severe injuries are correctly identified [13,26]. This in turn can prompt a sense of capability, competency and thus motivation to work with wildlife.

### Collaboration versus Conflicts

This research explored three areas of collaboration. Within the veterinary community, with wildlife centres and with the public. However a lack of collaboration and poor communication can lead to conflicts and discord which was also described.

The veterinary interviewees outlined several ways they offered or received wildlife support from their colleagues and peers, by sharing information, training and developing a network of contacts. This inter-veterinary support was highlighted by many survey respondents who used colleagues and peers as a wildlife resource. These supportive relationships between practices and within a team, as either a top down and bottom-up approach, offered a positive working environment and attitude towards wildlife and a sense of unity. Several instances offered by the interviewees of either themselves leading the team by example or management setting a precedent and creating a wildlife legacy. This approach leads to a level of autonomy and feeling of empowerment to embrace the challenge and make decisions [25,27]. In particular veterinary nurses who can be the best resource for evidence-based information, problem-solving stratagem, support and guidance when it comes to wildlife care [28,29]. Collaborations also extended to others who work with wildlife, predominantly wildlife rehabilitation centres. Two-thirds of survey participants highlighted wildlife centres as an information resource and only 8% had no relationship or communication with a wildlife centre. Both veterinary and wildlife centre interviewees highlighted the benefits of these mutual relationships; sharing of information, the goal of best care strategies, sharing the workload and patient transfers, similar to those found by Haering et al.[8] A collaborative relationship between veterinary clinics and wildlife centres was advocated by Mullineaux and Haering *et al.* to provide and facilitate high levels of care, rehabilitation and treatment options [8,19]. Veterinary professionals that work, volunteer or complete training at a wildlife centre help to directly consolidate those collaborative relationships.

Unfortunately, both veterinary and wildlife interviewees described a range of conflicts. Veterinary interviewees described experiencing judgement, contempt and resistance from colleagues and peers with negative opinions towards wildlife casualties, particular if a ‘vermin’ perception was involved and a lack of ‘sharing the load’ when colleagues and peers were reluctant or refused to treat wildlife. Issues between veterinary and wildlife organisations included poor communication, differing opinions for treatment, prolonged and variable triage, reluctant or refusal to see wildlife and convenience euthanasia. Barnes found that only 35% of veterinary practices felt they had reliable support from an external wildlife organisation [2], and 13% of the survey participants believed their relationship with a wildlife centre could be better. Whereas Haering et al*.* found only 50% of surveyed wildlife centres felt their local veterinarian understood wildlife assessment protocols and caused prolonged suffering due to delayed or with-holding euthanasia [14]. Evidence of delayed and minimal wildlife triage seems rife in the veterinary industry. Haering et al. and Orr and Tribe found only 26.2% and 20% of veterinary clinics respectively, examined wildlife cases immediately upon presentation [8,26]. In addition, Stidworthy et al. discussed wildlife that were not examined at all or received only cursory examinations often resulted in severe injuries being missed before being sent to a wildlife centre [11]. Delayed triages and missed injuries were issues of concern discussed at length by all the wildlife centre interviewees. These areas of conflict all lead to feelings of tension, frustration and mistrust which can culminate in arguments, welfare issues and more euthanasia outcomes.

Another area of collaboration and conflict is the public. The public can be a source of assistance to both veterinary clinics and wildlife centres by providing financial support, patient transfers, volunteer work and citizen science. Both veterinary and wildlife interviewees discussed the benefits of collaborating with the public including public relations, giving back to the community, improved practice reputations and public education. However, the survey data showed that although a third of respondents thought they should communicate with the public about wildlife work, only a third currently did. Reasons for this were offered by the interviewees. That despite the favourable benefits of publicising wildlife care [30], there was a risk of a snowball effect resulting in increased wildlife caseload to that practice causing a time, personnel and financial burden. This logic is supported by Haering et al. who found the public, responsible for 68% of all wildlife casualties entering the veterinary sector, would favour practices who actively treated wildlife and avoided those less willing or able to [8]. In addition to snowballing caseloads, other reasons why the final third of survey respondents did not communicate wildlife work to the public were unrealistic public expectations, discussed by both veterinary and wildlife centre interviewees. These expectations ranged from; all veterinary clinics being eager and capable of treating wildlife with free unrealistic, unlimited and irresponsible treatments, also voiced by Barnes and Cooper and Cooper [2,30], to veterinarians only euthanase wildlife. Often discussions about euthanasia for prognosis or welfare reasons were met with hostility and a potential for social media backlash. The relationship with the public is therefore complicated. The public can have an invested interest in wildlife and be a strong ally in wildlife work. However, with increased demand for wildlife services, emotive unrealistic expectations and social media criticism, an imbalance between public presumptions and clinical realities can accumulate.

### Regulatory Framework

The RCVS is the regulatory body for veterinary professionals in the UK. They are responsible for setting the standards of veterinary education and setting and regulating the professional standards.

During the interviews, conversations concerning wildlife standards in the CPCs produced two main responses. Either that the Codes were adequate guidelines for the veterinary profession, or that the Codes were ambiguous and open to interpretation. The VPS respondents ranked Obligations and the RCVS code as the second highest influential factor to treat wildlife (Fig 3), suggesting an understanding of the professional obligations to wildlife. However an example of the RCVS CPC that could be subject to misinterpretation is Article 1.2 states *A veterinary surgeon or nurse must restrict their dealings with animals to their area of expertise* [5,6]. This offers a justification or excuse to a veterinary surgeon or nurse to avoid wildlife casualties if they deem wildlife is not their area of expertise. The RCVS acknowledged the potential for misinterpretation and confusion when they released an online course in 2023; “*Understand your role in dealing with and treating wildlife*” [31] and a Standards and Advice Update [32] in 2024. Both reiterated the obligations of the professional to provide veterinary care to wildlife casualties.

Despite these obligations, wildlife is not explicitly discussed within the educational standards or day one competencies for veterinarians or veterinary nurses [33–36], This was confirmed unanimously by the educator interviewees and a word search for ‘wildlife’ and synonymous words within these documents produced zero results. Therefore, there is a disparity between the expectations of the RCVS on the veterinary profession to provide care to wildlife [3,29] without the assertion that they will receive the education in order to do so.

Those professionals who do not act within the CPCs are subject to regulation by the RCVS. Interestingly, over half of the survey respondents were not aware that the RCVS regulated wildlife complaints, and the topic yielded disdainful responses from both veterinary and wildlife interviewees about the realities of the regulation. In contrast Mullineaux and Stanford described increasing numbers of wildlife litigation cases made to the RCVS against veterinary professionals including delayed or refusal to see wildlife casualties, negligence, poor standard of care, welfare issues and prescription issues [13,37]. Stanford also reported that wildlife litigation cases defended by the Veterinary Defense Society were increasing [37]. However a search for ‘wildlife’ and synonymous words in the litigation cases published online by the RCVS [38] from 2022 to 2024 yielded zero results and the RCVS were unable to provide any information for this research when directly approached.

The current mixed understanding of the RCVS CPCs towards wildlife, lack of educational standards and hazy regulation of wildlife complaints suggests that the RCVS could do more to improve the treatment of wildlife by the veterinary community.

### Limitations

We acknowledge several limitations with this study. The survey and interview sample sizes are too small to be considered representative of their communities; with over 53,500 UK-practicing veterinary surgeons and veterinary nurses [39], over 700 wildlife rescuers [40], 9 veterinarian universities [41] and 76 veterinary nursing universities and colleges in the UK at the time of publication [42]. Although recruitment achieved data saturation for both veterinary professionals and wildlife centres personnel, there was data scarcity in the academic educator group.

The convenience nature of sampling could result in a participant bias in both the survey and interviews with veterinary professionals interested in wildlife being more likely to respond to recruitment. Despite an open recruitment strategy to encourage participants with alternative views to participate. This could lead to the absence of alternative characteristics [43], including those working in alternative fields or roles within the veterinary industry or those resistant or reluctant to treat wildlife. There is also the potential of social desirability bias by the participants, predominantly in the face-to face interviews [43].

The survey data has only been analysed by descriptive analysis with no inferential statistics which limits the results, and ideally the interviews would be analysed by several coders to improve reliability and accuracy [43]. Being an observational qualitative study with the potential for participant and researcher bias could also result in limitations to reproduce identical data both in interview performance and evaluation [43]. Finally this research is also UK-based and thus could be limited in its relevance or application internationally.

### Pathways for Wildlife Engagement

There are several ways that this research can be applied to improve the care of wildlife casualties in veterinary practice.

On a practice level, by involving the whole team in discussions regarding wildlife obligations and care, personnel are given the opportunity to either enhance and promote their intrinsic motivators [25] or discuss their concerns and challenges. Role assignment, wildlife protocols and information resources would improve competency, compliance, responsibility and accountability, whilst simultaneously reducing limiting factors like time, cognitive load, and anxiety of the unknown. Communication and support within the team and from management could convert the prospect of wildlife casualties from an obligational burden to a fulfilling experience.

On a professional level, collaborations with both veterinary peers and wildlife centres can improve wildlife care and welfare and help share the load of wildlife casualties. By developing networks for support, advice and the exchange of knowledge, facilities and experience. The benefits of then communicating these activities and relationships to the public include improved public relations for both the clinic and the general veterinary profession and the opportunity to improve public understanding, education and acceptance.

At an educational level, veterinary schools can acquaint their unsuspecting students of the high probability of seeing wildlife in their career [10,19] and provide fundamental skills and knowledge to guide them. From adapting first principles and utilising resources to public communication and work experience with wildlife hospitals.

The RCVS has the potential to incorporate and facilitate wildlife medicine in the veterinary community. The explicit inclusion of wildlife into the Professional Code of Conduct and improve the regulation of wildlife complaints. Set a consistent benchmark for wildlife medicine and care in the veterinary and veterinary nurse education standards and day one competencies and cultivate relationships with wildlife organisations like the British Wildlife Rehabilitation Committee to help determine ways to bridge the relationship between the wildlife and veterinary industries.

## Conclusions

The key findings of this research are that with improved education from veterinary and veterinary nursing schools, continued in-house training, endorsement and encouragement within and between veterinary practices and wildlife centres, and with support from the RCVS, wildlife work could be embraced rather than possibly dreaded. Working together to improve communication, utilise resources, mitigate inhibiting factors and develop networks would lead to the benefits of improved skills, personal and team achievements and pragmatic connections with the community. There is the added potential to counterbalance some of the losses to wildlife populations and diversity caused by anthropogenic factors and improve wildlife welfare and medicine.

## Supporting information

S1 Table Interview codes, subthemes and themes using thematic analysis. (DOCX)

S2 Document The interview scripts for veterinary professionals, wildlife centre personnel and academic educators. (DOCX)
