# Supplementary material for: Influences on veterinary professionals to perform wildlife medicine in the United Kingdom: A qualitative multifocal study
Source: PLoS One. 2026 Oct 27;21(10):e0341752. doi: 10.1371/journal.pone.0341752 (PMC13712581; doi:10.1371/journal.pone.0341752)
Supplement: Supplementary file 1 [file pone.0341752.s004.pdf]

PONE-D-26-00221R1

1. Overall Assessment

Major weaknesses:

- **Participant bias** adequately acknowledged but not fully addressed – the sample likely overrepresents wildlife-interested professionals.
- **Survey analysis is purely descriptive** – no statistical testing or inferential analysis, limiting its contribution beyond describing the sample.
- **Results and Discussion are combined** – while acceptable for qualitative work, this sometimes makes it difficult to distinguish raw findings from interpretation.
- **Limited generalizability** acknowledged but the small interview sample (especially n=7 for wildlife centres) may not have reached full saturation.
- **Some overclaiming** – e.g., "will require a system change" (Abstract) is strong language for a qualitative study of 409 + 33 participants.

---

2. Criteria-Based Review

A. Clarity and language

- **Acceptable with minor edits.**
- Abstract is clear and well-structured.
- **Issues:**
    - Title is overly long and repetitive – consider shortening (e.g., *"Influences on UK veterinary professionals' engagement with wildlife medicine: A qualitative study"*).
    - Some sentences are overly complex (e.g., lines 87–90, 109–112) – simplify for readability.

- Typographical: “promotors” (line 224) should be “promoters” (consistent with Abstract uses “promoters”).
- Fig 2, 3, 5 – word clouds are visually appealing but less informative than bar charts with percentages.
- Some references missing page numbers or DOIs (e.g., ref 4, 19, 20).

B. Originality and significance

- **Originality: Moderate-high** – This is the first qualitative UK study to systematically explore both promoters and inhibitors of wildlife treatment across multiple stakeholder perspectives (vets, nurses, wildlife centres, educators).
- **Significance: High** – Wildlife populations are declining; veterinary professionals are critical first responders. The findings have direct implications for RCVS policy, veterinary education curricula, and practice-level protocols.
- **Novel contributions:**
  - Explicit identification of RCVS as a central (but currently underutilized) influence.
  - Species bias mapping (hedgehogs most favored, pigeons/rats most disfavored).
  - GRPI model application to wildlife medicine in practice.

C. Methods (PLOS ONE key area)

Criterion	Present?	Quality
Ethical approval	Yes (HERC_2024_120)	Excellent
Informed consent	Yes (written)	Excellent
Survey recruitment strategy	Clearly described	Good

Criterion	Present?	Quality
Interview recruitment	Described per group	Good
Sample size justification	Partial (cited 12/saturation but only 7 wildlife centre interviews)	Needs clarification
Data saturation statement	Not explicitly stated	Should add
Thematic analysis approach	Braun & Clarke (2006) – clear	Good
Positionality statement	Yes (line 182–187)	Excellent
Data availability	Full transcripts + survey deposited	Excellent (exemplary)
Pilot interviews	Yes	Good

Critical improvements needed:

1. **Saturation:** The authors state they aimed for "at least twelve interviews per group" (line 146) but only achieved 20 (vets), 7 (wildlife centres), and 6 (educators). This needs justification – e.g., did data saturation occur for vets? Was it not achieved for wildlife centres/educators, and how does this limit findings?
2. **Survey analysis:** The survey is described as "descriptive rather than statistical" (line 139). However, with n=409, basic inferential tests (chi-square, logistic regression) could have identified associations (e.g., between education level and confidence, or practice type and wildlife protocols). This is a missed opportunity.
3. **Interview guide availability:** The author's state templates are available at the Data Share link, but they are not included in the manuscript. Recommend including as Supplementary File S1.

4. **Coding reliability:** Only one researcher coded the data. While positionality is acknowledged, inter-coder reliability or researcher triangulation would strengthen validity. Consider adding a statement about reflexive practices.

D. Results

Weaknesses:

1. **No inferential statistics** – Table 2 shows percentages but no tests of association. For example:
   - Is wildlife CPD uptake associated with practice type?
   - Is awareness of RCVS investigations associated with years since graduation?
   - These analyses would strengthen the paper.
2. **Results and Discussion combined** – While acceptable for qualitative research, it makes it harder to trace the raw data to the interpretation. Consider separating into distinct sections or clearly signposting when interpretation begins.
3. **Lack of negative cases** – The authors acknowledge participant bias (line 203–207) but do not explore whether any interviewees expressed *negative* or *reluctant* views towards wildlife. This would have strengthened the analysis.
4. **Quantitative data not fully integrated** – The survey and interview data are presented separately; a mixed-methods integration (e.g., joint display) would enhance the narrative.

E. Discussion

Problems:

- **Overclaiming** – "will require a system change" (Abstract) is too strong for a qualitative study of 409 + 33 participants. Suggest "may benefit from" or "would be supported by."
- **Long-term sustainability not addressed** – The recommendations (e.g., protocols, CPD) are plausible but no evidence of feasibility or cost-effectiveness is provided.
- **No limitations section** – This is a significant omission. PLOS ONE requires authors to discuss limitations. Add a dedicated subsection covering:
  - Participant bias (self-selection).
  - Small wildlife centre/educator sample.

- Single coder.
- No inferential statistics.
- Potential social desirability bias in interviews.
- Limited generalizability beyond the UK.

F. Ethical standards & data availability

- **Ethics:** Excellent – HERC approval, written informed consent.
- **Data availability: Exemplary** – Full survey results, interview scripts, and transcripts deposited at Edinburgh DataShare (DOI: 10.7488/ds/8059). This exceeds PLOS ONE requirements.

Minor suggestion: Clarify that interview transcripts were anonymized before deposition.

G. References

Issues:

- Ref 4 (Barnes) – available as a thesis; should include DOI or URL.
- Ref 19 (Ando et al., 2014) – page numbers missing.
- Ref 20 (Guest et al., 2006) – page numbers missing.
- Some references (e.g., 26, 27, 28) are web-based; include access dates (some have them, some don't). Standardize.

---

3. Major Required Revisions (for publication)

1. **Add a dedicated Limitations section** (see E above for content).
2. **Clarify data saturation** – explicitly state whether saturation was achieved for each stakeholder group; if not, acknowledge this as a limitation.
3. **Consider adding inferential statistics** to the survey analysis (at least chi-square tests for key associations) or justify why they are omitted.
4. **Separate Results and Discussion** or clearly delineate when interpretation begins (e.g., “This suggests that…”).
5. **Tone down overclaims** – replace “will require” with “would benefit from” or “may require” (Abstract and Conclusions).
6. **Add interview guides as Supplementary File S1** (or confirm they are at the DataShare link).

---

4. Minor Revisions

- Typo: “promotors” → “promoters” (line 224).
- Title: shorten to *“Influences on veterinary professionals to perform wildlife medicine in the United Kingdom: A qualitative study”* (or similar).
- Fig 2: Bar chart with percentages would be more informative than the current unlabelled ordinal ranking.
- Fig 3 and 5: Word clouds are engaging but should be accompanied by a frequency table in the text.
- Line 87–90: Simplify the long sentence about the Canadian policy.
- Line 492–493: Clarify whether “10%” and “7%” refer to survey respondents or interview mentions.
- Standardize access dates for all web references.
- Check all abbreviations: SOPs (standard operating procedures) defined at line 573, but “CPD” not defined until line 287 – define on first use (line 115 or earlier).
