# Supplementary material for: Influences on veterinary professionals to perform wildlife medicine in the United Kingdom: A qualitative multifocal study
Source: PLoS One. 2026 Oct 27;21(10):e0341752. doi: 10.1371/journal.pone.0341752 (PMC13712581; doi:10.1371/journal.pone.0341752)
Supplement: Supplementary file 2 [file pone.0341752.s001.docx]

**S1 Table: Interview codes, subthemes and themes using thematic analysis**

| Themes | Subthemes | Codes |
| --- | --- | --- |
| Promotors |  |  |
| Collaborations | Veterinary community | Personnel - team work |
|  |  | Peer support within and between clinics |
|  |  | Practice culture and legacy - management |
|  |  | Mentoring students |
|  | Wildlife centres | Support and advise from wildlife centres |
|  |  | Sharing knowledge, information and resources |
|  |  | Wildlife centre training courses |
|  | Public relations | Providing a community service |
|  |  | Share the load for transfers and rehabilitation |
|  |  | Emotive citizen involvement |
|  |  | Public education and explanations |
| Competency | Knowledge | Education – absent or no recollect |
|  |  | Education - Core or elective or combined |
|  |  | Combined with experience & exposure |
|  | Training/CPD | Online or hands on training with wildlife centres |
|  |  | External qualification in wildlife +/- exotics |
|  |  | Wildlife lectures at veterinary conferences |
|  | Experience | Collaborations with wildlife hospitals |
|  |  | Volunteering with wildlife organisation |
|  |  | Small animal vs large animal practitioners |
| Motivation | Positive species bias | Exposure to specific species |
|  |  | Area specific species (red squirrels) |
|  |  | Population effects on wildlife |
|  | Interest/passion | Concerns about welfare |
|  |  | Wildlife advocate |
|  |  | Personal and team effect of positive outcomes |
|  |  | Population effects on wildlife |
|  | Volunteering | Providing wildlife CPD or advice on online |
|  |  | Transfers between veterinary and wildlife centres |
|  |  | Volunteering with wildlife organisation |
|  |  | Having own backyard rehabilitation set up |
| Inhibitors |  |  |
| Conflicts | Wildlife centres (WC) | Backyard rehabbers providing inappropriate care |
|  |  | WC not responding to case or training requests |
|  |  | Unable to contact WC about transfers or advice |
|  | Veterinary community | Colleagues or management blocking wildlife care |
|  |  | Clinics refusing or reluctant to see wildlife |
|  |  | Not sharing the load |
|  | The public | Interfering with wildlife creates additional work |
|  |  | Public assumption that veterinarians just euthanase |
|  |  | Public expectations that veterinarians can save everything |
|  |  | Snowball effect of accepting wildlife cases |
| Uncertainty | Species bias | Overrepresented in wild / brought to clinics |
|  |  | Considered vermin |
|  |  | Transmission of disease |
|  |  | Risk of bites, scratches or kicks |
|  | Lack of Knowledge | Absent or poor university education |
|  |  | Student un-appreciation for wildlife work |
|  |  | Species ecology and biology |
|  |  | Time and cost of continued learning |
|  |  | Lack of exposure, experience and confidence |
|  |  | Unknown ethics and facilities of local wildlife centres |
|  | Consensus | Veterinary community consensus for treatments |
|  |  | Rehabilitator consensus for treatments |
|  |  | Lack of standards within wildlife centres |
| Lack of Resources | Time | Busy clinics / consultations |
|  |  | Lack of personnel for cleaning, feeding and care |
|  | Facilities | Lack of kennel space or areas. |
|  |  | Wildlife near predator species (dog/cat) |
|  | Protocols & SOPs | Absent protocols or SOPs |
|  |  | Unread or under-utilised protocols or SOPs |
|  | Finances | Unknown financial allocation for wildlife |
|  |  | Financies are consideration but not an inhibitor |
|  |  | Don’t ask or not told no |
| RCVS | Professional code of conduct | Sufficient Code of Conduct |
|  |  | Ambiguous and misinterpretation |
|  | Regulation | Regulation is adequate |
|  |  | Regulation is inadequate |
|  | Education standards | Day one skills and basics principals |
|  |  | Lack of educational standards |
| Outcomes | Lack of triage | Clinics refusal to see wildlife |
|  |  | Clinics reluctant to see wildlife |
|  | Inappropriate triage | Lack of knowledge, time, experience or confidence |
|  |  | Species bias |
|  | Inappropriate Euthanasia | Lack of knowledge & confidence |
|  |  | Lack of rehabilition or support |
|  |  | Lack of time, facilities or finances |
|  |  | Wildlife or species bias |
