# Supplementary material for: Influences on veterinary professionals to perform wildlife medicine in the United Kingdom: A qualitative multifocal study
Source: PLoS One. 2026 Oct 27;21(10):e0341752. doi: 10.1371/journal.pone.0341752 (PMC13712581; doi:10.1371/journal.pone.0341752)
Supplement: Supplementary file 3 [file pone.0341752.s002.docx]

**Veterinary Professional Interview Script**

1. Tell me where you are in the UK, the type of wildlife you see and the status of wildlife in your area.
2. How of you feel about treating wildlife, how confident are you?
3. What factors encourage or enable you to treat wildlife cases?
4. What factors create a challenge for you treating wildlife?
   1. Health and safety and disease risk
   2. Colleagues/employees
   3. The public
5. What protocols and SOPS do you have at your clinic regarding wildlife?
6. Does your practice communicate with clients/public about wildlife patients?
7. Tell me about your relationship with local wildlife centres?
8. Could the wildlife centre offer or provide anything to help you treatment of wildlife?
9. Over the past 50 years, wildlife numbers in the United Kingdom have dropped dramatically by 19% and continue to decrease. Do you think the veterinary community as a whole can contribute to reducing wildlife loss?
10. Do you think wildlife medicine should be included in veterinary university’s curriculum?
11. Do you think the RCVS should get involved in encourage the veterinary community to treat more wildlife cases?
1. Is there anything else you would like to add?

**Wildlife Centre Personnel Interview Script**

2. Tell me about the wildlife centre and your position there?
3. Tell me about the wildlife you see and how they receive medical treatment?
4. What are the best and worst animals to treat and rehabilitate and why?
5. What's your general impression about the knowledge and skills that veterinary professionals possess regarding wildlife?
6. Describe your relationship with local veterinary clinics and the main reasons for contact between clinics and centre
7. Do you have a relationship with local wildlife centres?
8. What about the public, Are they a help or a hinderance?
9. How would you feel about the following:
   1. Having a out-of-hours phone contact for veterinary practices only, seeking advise, sending photos and videos.
   2. Providing feedback about cases from veterinary practice either regarding medical successes or case management suggestions?
   3. Accepting on-site visits from local veterinary professionals to see the facilities and discuss a collaborative relationship?
   4. Providing training/CPD regarding handling, husbandry, basic care
10. Over the past 50 years, wildlife numbers in the United Kingdom have dropped dramatically by 19% and continue to decrease. What role could veterinary practices and wildlife centre play to reduce this loss?
11. Are you aware of the Royal College of Veterinary Surgeons and the Professional Code of Conduct that sets the standards for veterinary professions?
12. Is there anything else you would like to add?

**Academic educator interview Script**

13. Is wildlife medicine part of the core education, an elective or both in the veterinary undergraduate curriculum?
14. When and why did wildlife medicine become part of the curriculum?
15. At what stage/year of education is wildlife medicine taught?
16. Can you provide in brief the details regarding individual and population wildlife medicine taught
17. Do you use wildlife cadavers for anatomy learning?
18. Would you consider wildlife patients to be a day one competencies that new-graduates will be expected to treat when entering the profession?
19. Do you think the RCVS should specify basic wildlife triage medicine in the accreditation standards and as a day one competency?
20. Would you consider having EMS at a wildlife rehabilitation centre a mandatory placement?
21. What is the general feedback from students about wildlife medicine as part of the curriculum?
22. What is your opinion on wildlife medicine as part of the curriculum?
23. Is there anything else you would like to add?
