# Supplementary material for: Influences on veterinary professionals to perform wildlife medicine in the United Kingdom: A qualitative multifocal study
Source: PLoS One. 2026 Oct 27;21(10):e0341752. doi: 10.1371/journal.pone.0341752 (PMC13712581; doi:10.1371/journal.pone.0341752)
Supplement: Supplementary file 7 [file pone.0341752.s007.docx]

**Influences on veterinary professionals to perform wildlife medicine in the United Kingdom**

**Critical Evaluation:**

This study investigated how culture, education, professional obligations, and working relationships influence how veterinary professionals in the United Kingdom treat wildlife. Using a mixed-methods approach, researchers collected data from 409 veterinary survey respondents and conducted interviews with veterinary professionals, wildlife centre personnel, and academic educators. The findings identified three key factors that promote wildlife treatment competency, motivation, and collaboration among stakeholders. Major barriers included uncertainty, insufficient resources, and conflicts within the veterinary and wildlife rehabilitation sectors. The study concluded that improving wildlife medicine education, strengthening collaboration between veterinary practices and wildlife centres, and enhancing regulatory and professional support from the Royal College of Veterinary Surgeons (RCVS) could foster a stronger wildlife medicine culture in the UK.

**Critique:**

The manuscript is well written, addresses an important one-health issue and has relevant findings. However, one major limitation of the study is its use of **purposive and convenience sampling**, which may introduce selection bias and limit the generalisability of the findings. Participants with a particular interest in wildlife medicine may have been more likely to respond, meaning the results may not fully represent the views and experiences of the wider veterinary profession in the United Kingdom.

**Recommendation**: The study can be approved
