# Supplementary material for: Influences on veterinary professionals to perform wildlife medicine in the United Kingdom: A qualitative multifocal study
Source: PLoS One. 2026 Oct 27;21(10):e0341752. doi: 10.1371/journal.pone.0341752 (PMC13712581; doi:10.1371/journal.pone.0341752)
Supplement: Supplementary file 8 [file pone.0341752.s003.docx]

**Dear the ACADEMIC EDITOR:**

Please find below the responses to your edits. Line numbers are in relation to the new Manuscript document.

**Abstract; please arrange the abstract with the headings and as stated below:**

- **Introduction/background: (Objective(s) of the study clearly stated):** Heading has been added (line 23) and objectives have been described (lines 25-29).
- **Methods: Ensure that you mention qualitative and quantitative analysis, sampling method (purposive?) for qualitative analysis and the number of participants.** Heading (line 30) and clarification of the qualitative and quantitative data analysis (line 32 & 35), sampling methods (line 31 & 33). The number of interview participants was already stated but hopefully now clearer (line 33-34)
- **Procedures to achieve results:** unsure what additional information you are asking for that are not in the methods?
- **Results** Heading added (line 36)
- **Conclusion** Heading added (line 41)

**Introduction; please ensure that you arrange your facts sequentially as stated below:**

- **Global state of wildlife extinction:** Global mention of wildlife extinction has been removed.
- **Services –** professional obligations have been moved to lines 64-73
- **Major challenges -** have been rearranged; unknown wildlife admission numbers, professional obligations, current inhibiting factors, wildlife centre relationships.
- **Continental state of wildlife and veterinary services:** European wildlife levels and veterinary services are no relevant in this UK based project.
- **What is obtainable in the UK with regards to wildlife veterinary services? –** This is part of the research question (line 97-100)
- **Challenges, research questions and objective of the study as the last statement of the last paragraph –** Clarified and is final paragraph Line 93-100

**Methods**

- **What were the semi structured questions? Please, include for clarity.** All three interview scripts are available on the data share DOI I have clarified this on line 134
- **LINE 125; state the meaning of GDPR before abbreviating –** Sentence including GDPR has been removed.
- **What type of sampling technique was used in the selection of your study participants? Please, state clearly.**
- Survey and interview sampling were both purposive, convenience sampling now stated on line 108 and 127.
- Interview quota aim was 12 with a max of 20 interviews per group, now stated on line 130-132 & 136-137.
- **Ensure to rephrase this statement lines 170-172” Each interview was recorded audibly and transcribed by Microsoft Teams software during the interview, with post editing for accuracy and verbatim“. -** Rephased on lines 150-152
- **State the type of device you used in recording** Online using a laptop (line 150)
- **The average time for the interviews conducted.** Already in the manuscript on line 152.
- **The statement on data saturation was not clear, state clearly whether data saturation point was attained during the interviews? Was there a point you felt that there was no new information generated during the interviews?** Data saturation was achieved for the veterinary professional group but not for the wildlife centre and academic educator groups. Clarification on lines 136 and 145-147.

**Results**

**Construct a table that indicates codes generated, subthemes and themes that emerged from the thematic analysis for a broader perspective of what was done and clarity. –** Table created and in Supportive Material – S1 Table Line 228 & 878

**Line 238; ensure that you write veterinarian in full in the translation for clarity –** corrected to veterinarian(s) or veterinary in all the quotes

**The quantitative analysis augments the findings of the qualitative analysis or may even defer. While I agree with the potency of the study. There should not be discussion in the results. Clearly state your findings on the survey (quantitative analysis) separately. Then, state your findings in the qualitative analysis.  Finally in the DISCUSSION section you can compare and contrast your findings. This is essential for clarity. –** Results and Discussion have been separated.

**Line 286; write the full meaning of BVA. –** sentence including BVA has been removed.

**Write the full meaning of CPD before abbreviating. –** Written in full (continual professional development) at the first mention of CPD in Table 2.

**Line 314; detrimental effects on students not “of students”. -** corrected

**Uni? Please, write university in full. –** corrected all “uni” to “university”

**Your findings should be the basis of your results. Discussions on the findings and your opinions should be strictly related in the discussion section. –** Results and Discussion have been separated

**Ensure that you write this abbreviation in full “RSPCA”. –** RSPCA has been removed

**Line 425; ensure that you remove referencing from results “Mullineaux and Haering et al.” - –** Results and Discussion have been separated so references are only in discussion.

**Line 437; iSpy?** removed

**Discussion for this manuscript is basically absent which should not be so. Like I mentioned earlier, the survey results should be clearly stated, findings of the qualitative studies should be clearly stated under the themes and subthemes developed while discussions of the findings should be under discussion. –** Results and Discussion have been separated

**Furthermore, the last paragraph in the discussion should be the limitations of the study. –** Limitations moved to end of discussion line 815-823

I hope these edited are sufficient to your requests. Please let me know if I have misunderstood anything.

I look forward to hearing from you.

Rebecca Lang.
