# Supplementary material for: Influences on veterinary professionals to perform wildlife medicine in the United Kingdom: A qualitative multifocal study
Source: PLoS One. 2026 Oct 27;21(10):e0341752. doi: 10.1371/journal.pone.0341752 (PMC13712581; doi:10.1371/journal.pone.0341752)
Supplement: Supplementary file 9 [file pone.0341752.s005.docx]

PONE-D-26-00221R1

**Major Required Revisions (for publication)**

|  | Comment | Response |
| --- | --- | --- |
| 1 | Add a dedicated Limitations section (see E above for content).  o Participant bias (self-selection).- Participant bias adequately acknowledged but not fully addressed – the sample likely over-represents wildlife-interested professionals.  o Small wildlife centre/educator sample.  o Coding reliability: Single coder. While positionality is acknowledged, inter-coder reliability or researcher triangulation would strengthen validity. Consider adding a statement about reflexive practices.  o No inferential statistics.  o Potential social desirability bias in interviews.  o Limited generalizability beyond the UK. | Comment seems to have made in relation to Manuscript PONE-D-26-00221 uploaded on 11/01/2026, not the revised manuscript PONE-D-26-00221R1 updated on 23/04/2026.  A Limitations section was added and I have edited it further on line 813-835. |
| 2 | Saturation: The authors state they aimed for “at least twelve interviews per group” (line 146) but only achieved 20 (vets), 7 (wildlife centres), and 6 (educators). This needs justification – e.g., did data saturation occur for vets? Was it not achieved for wildlife centres/educators, and how does this limit findings?  Clarify data saturation – explicitly state whether saturation was achieved for each stakeholder group; if not, acknowledge this as a limitation. | Data saturation between the three groups of interviews has been hopefully described more clearly - line 150-156.  Also in Limitations - line 818-820. |
| 3 | Survey analysis is purely descriptive – no statistical testing or inferential analysis, limiting its contribution beyond describing the sample.  Consider adding inferential statistics to the survey analysis (at least chi-square tests for key associations) or justify why they are omitted. | As the survey sample size was small and it excludes the representation of university educators and wildlife centres I decided to prioritize the thematic analysis of the interviews to produce a richer, deeper qualitative study, which has not previously been performed or published in the UK. |
| 4 | Quantitative data not fully integrated – The survey and interview data are presented separately; a mixed-methods integration (e.g., joint display) would enhance the narrative. | The quantitative survey data is now mixed with the interview thematic results in the Results section - Line 178 - 542 |
| 5 | Results and Discussion are combined – while acceptable for qualitative work, this sometimes makes it difficult to distinguish raw findings from interpretation.  Separate Results and Discussion or clearly delineate when interpretation begins (e.g., “This suggests that…”). | Comment seems to have made in relation to Manuscript PONE-D-26-00221 uploaded on 11/01/2026, not the revised manuscript PONE-D-26-00221R1 updated on 23/04/2026.  The results and discussion were separated in the R1 version. Now Results section - Line 178 – 542, Discussion Section line 544 - 869 |
| 6 | Tone down overclaims – replace “will require” with “would benefit from” or “may require” (Abstract and Conclusions). | Changed as suggested on line 40 in the abstract and has been removed from the conclusion. |
| 7 | Add interview guides as Supplementary File S1 (or confirm they are at the DataShare link). | Interview scripts available as a supplement document S2 File on line 138 and at <https://doi.org/10.7488/ds/8059>. - |
| 8 | Lack of negative cases – The authors acknowledge participant bias (line 203–207) but do not explore whether any interviewees expressed negative or reluctant views towards wildlife. This would have strengthened the analysis. | Negative or reluctant views from the interviews were scarce but have been highlighted and included in the manuscript, mainly in the inhibitor results.   [Wildlife] is often something that you've never treated or potentially never handled before and I think it's just quite a daunting thing (VP18). Line 388-389.  “We just get so inundated with birds and fledglings. I have a really negative reaction to it… especially pigeons… the winged rats of the world (VP17). Line 403-405  We don't always have enough time to provide as proficient care [to wildlife] as we would for others (VP17). Line 426-427. |
| 9 | Long-term sustainability not addressed – The recommendations (e.g., protocols, CPD) are plausible but no evidence of feasibility or cost-effectiveness is provided. | Section called “Pathways for wildlife engagement” has been added. Line 837-869 |

**Minor Revisions**

| 1 | Typo: “promotors” → “promoters” | Updated 4 corrections: line 38, 201, 202, 551. |
| --- | --- | --- |
| 2 | Title is overly long and repetitive – consider shortening: | Changed to: “Influences on veterinary professionals to perform wildlife medicine in the United Kingdom. A qualitative multifocal study.” – I will email to change the title on data share link |
| 3 | Fig 2: Bar chart with percentages would be more informativ3 | Redone with percentages - now Fig 3. |
| 4 | Fig 3 and 5: Word clouds accompanied by a frequency table in the text. | Word clouds now accompanied by a percentage pie-chart - Renamed Fig 4 and Fig 5 |
| 5 | Lines 87–90 & 109–112: simplify for readability. | Sentences simplified - see line 69-72 and 95-97 |
| 6 | Line 492–493: Clarify whether “10%” and “7%” refer to survey respondents or interview mentions. | Clarified - see line 396-397 |
| 7 | Clarify that interview transcripts were anonymized before deposition. | Statement add - line 159-160 |
| 8 | References.  Ref 4 (Barnes) – available as a thesis; should include DOI or URL.  Ref 19 (Ando et al., 2014) – page numbers missing.  Ref 20 (Guest et al., 2006) – page numbers missing.  Some references (e.g., 26, 27, 28) are web-based; include access dates (some have them, some don’t). Standardize. | Reference numbers have now changed as per second uploaded manuscript D-26-00221R1.  Ref 2 (Barnes) – DOI added  Ref 15 (Ando et al., 2014) – only page numbers 1-7 available on online article.  Ref 16 (Guest et al., 2006) – page numbers 59–82 added.  I have doubled checked and all web-based references have accessed dates in the format of [cited 2002 Aug 12] as per the Submission Guidelines. |
| 9 | Check all abbreviations: SOPs (standard operating procedures) defined at line 573, but “CPD” not defined until line 287 – define on first use (line 115 or earlier). | Abbreviation CPD and SOP first used and defined in Table Two on page 11.  UK first used and defined on line 22.  RCVS first used and defined on line 38.  VPS first used and defined on line 107.  CPC first used and defined on line 511 |
