# Supplementary material for: Influences on veterinary professionals to perform wildlife medicine in the United Kingdom: A qualitative multifocal study
Source: PLoS One. 2026 Oct 27;21(10):e0341752. doi: 10.1371/journal.pone.0341752 (PMC13712581; doi:10.1371/journal.pone.0341752)
Supplement: Supplementary file 10 [file pone.0341752.s006.docx]

**PONE-D-26-00221R2**

**1. Duplicate Content in Results Section**

**Location:** Lines 189–198 and Lines 248–253

**Issue:** The Results section contains **duplicate** information about the survey demographics and interview framework. This is a significant redundancy that should be corrected.

**First instance (lines 189–198):**

*"The online survey was completed by 409 veterinary professionals. The results were summarized in Table 1 and 2 and described in combination with the key informant interviews. The interviews were completed with twenty veterinary professionals, seven wildlife centre personnel and six university educators. Codes, subthemes and themes were developed using thematic analysis (S1 Table). A conceptual framework (Fig 1) illustrates three themes that promote engagement with wildlife treatment: Competence, Motivation and Collaborations. Three themes that inhibits engagement with wildlife: Uncertainty, Lack of resources and Conflicts, and a central influence by the RCVS."*

**Second instance (lines 248–253):**

*"Interviews were completed with twenty veterinary professionals, seven wildlife centre personnel and six university educators. Codes, subthemes and themes were developed using thematic analysis (S1 Table). A conceptual framework (Fig 5) illustrates three themes that promote engagement with wildlife treatment. Competence, Motivation and Collaboration. Three themes that inhibit engagement with wildlife. Uncertainty, Lack of resources and Conflict, and a central influence by the RCVS."*

**Recommendation:** **Delete the second instance (lines 248–253)** entirely. The first instance already introduces the framework, S1 Table, and the six themes. Keeping both is repetitive and unnecessary.

**2. Figure Number Inconsistency**

**Issue:** The conceptual framework is referred to by different figure numbers in different places:

| Location | Figure Reference |
| --- | --- |
| Results (first instance, line 194) | **Fig 1** |
| Results (duplicate instance, line 252) | **Fig 5** |
| Discussion (line 551) | **Fig 1** |
| Discussion (line 609) | **Fig 15** |

**Recommendation:** Unify all references to **Fig 1** for the conceptual framework. After deleting the duplicate instance, ensure all cross-references point to Fig 1.
