# Supplementary material for: Influences on veterinary professionals to perform wildlife medicine in the United Kingdom: A qualitative multifocal study
Source: PLoS One. 2026 Oct 27;21(10):e0341752. doi: 10.1371/journal.pone.0341752 (PMC13712581; doi:10.1371/journal.pone.0341752)
Supplement: Peer review history

# Editor report: Decision Letter 0

**DOI:** 10.1371/journal.pone.0341752.r001

**Authors:** Ayi Vandi Kwaghe

16 Feb 2026

Dear Dr. Lang,

Thank you for submitting your manuscript to PLOS ONE. After careful consideration, we feel that it has merit but does not fully meet PLOS ONE’s publication criteria as it currently stands. Therefore, we invite you to submit a revised version of the manuscript that addresses the points raised during the review process.

==============================

**ACADEMIC EDITOR:**

Abstract; please arrange the abstract with the headings and as stated below:

Introduction/background: (Objective(s) of the study clearly stated)

Methods: Ensure that you mention qualitative and quantitative analysis, sampling method (purposive?) for qualitative analysis and the number of participants.

Procedures to achieve results

Results

Conclusion

Introduction; please ensure that you arrange your facts sequentially as stated below:

Global state of wildlife extinction / services and major challenges

Continental state of wildlife and veterinary services

What is obtainable in the UK with regards to wild life veterinary services? Challenges, research questions and objective of the study as the last statement of the last paragraph.

Methods

What were the semi structured questions? Please, include for clarity.

LINE 125; state the meaning of GDPR before abbreviating

What type of sampling technique was used in the selection of your study participants? Please, state clearly.

Ensure to rephrase this statement lines 170-172” Each interview was recorded audibly and transcribed by Microsoft Teams software during the interview, with post editing for accuracy and verbatim“.

State the type of device you used in recording and the average time for the interviews conducted.

The statement on data saturation was not clear, state clearly whether data saturation point was attained during the interviews? Was there a point you felt that there was no new information generated during the interviews?

Results

Construct a table that indicates codes generated, subthemes and themes that emerged from the thematic analysis for a broader perspective of what was done and clarity.

Line 238; ensure that you write veterinarian in full in the translation for clarity.

The quantitative analysis augments the findings of the qualitative analysis or may even defer. While I agree with the potency of the study. There should not be discussion in the results. Clearly state your findings on the survey (quantitative analysis) separately. Then, state your findings in the qualitative analysis.  Finally in the DISCUSSION section you can compare and contrast your findings. This is essential for clarity.

Line 286; write the full meaning of BVA.

Write the full meaning of CPD before abbreviating.

Line 314; detrimental effects on students not “of students”.

Uni? Please, write university in full.

Your findings should be the basis of your results. Discussions on the findings and your opinions should be strictly related in the discussion section.

Ensure that you write this abbreviation in full “RSPCA”.

Line 425; ensure that you remove referencing from results “Mullineaux and Haering et al.”

Line 437; iSpy?

Discussion for this manuscript is basically absent which should not be so. Like I mentioned earlier, the survey results should be clearly stated, findings of the qualitative studies should be clearly stated under the themes and subthemes developed while discussions of the findings should be under discussion. Furthermore, the last paragraph in the discussion should be the limitations of the study.

Please ensure that your decision is justified on PLOS ONE’s publication criteria and not, for example, on novelty or perceived impact.

==============================

Please submit your revised manuscript by Apr 02 2026 11:59PM. If you will need more time than this to complete your revisions, please reply to this message or contact the journal office at plosone@plos.org. When you're ready to submit your revision, log on to https://www.editorialmanager.com/pone/ and select the 'Submissions Needing Revision' folder to locate your manuscript file.

- A letter that responds to each point raised by the academic editor and reviewer(s). You should upload this letter as a separate file labeled 'Response to Reviewers'.
- A marked-up copy of your manuscript that highlights changes made to the original version. You should upload this as a separate file labeled 'Revised Manuscript with Track Changes'.
- An unmarked version of your revised paper without tracked changes. You should upload this as a separate file labeled 'Manuscript'.

If you would like to make changes to your financial disclosure, please include your updated statement in your cover letter. Guidelines for resubmitting your figure files are available below the reviewer comments at the end of this letter.

If applicable, we recommend that you deposit your laboratory protocols in protocols.io to enhance the reproducibility of your results. Protocols.io assigns your protocol its own identifier (DOI) so that it can be cited independently in the future. For instructions see: https://journals.plos.org/plosone/s/submission-guidelines#loc-laboratory-protocols. Additionally, PLOS ONE offers an option for publishing peer-reviewed Lab Protocol articles, which describe protocols hosted on protocols.io. Read more information on sharing protocols at https://plos.org/protocols?utm_medium=editorial-email&utm_source=authorletters&utm_campaign=protocols.

We look forward to receiving your revised manuscript.

Kind regards,

Ayi Vandi Kwaghe, D.V.M., M.V.Sc., P.G.D.E. Ph.D., MPH, FETP

Academic Editor

PLOS One

**Journal Requirements:**

When submitting your revision, we need you to address these additional requirements.

1. Please ensure that your manuscript meets PLOS ONE's style requirements, including those for file naming. The PLOS ONE style templates can be found at

https://journals.plos.org/plosone/s/file?id=wjVg/PLOSOne_formatting_sample_main_body.pdf and

https://journals.plos.org/plosone/s/file?id=ba62/PLOSOne_formatting_sample_title_authors_affiliations.pdf

2. Please include a complete copy of PLOS’ questionnaire on inclusivity in global research in your revised manuscript. Our policy for research in this area aims to improve transparency in the reporting of research performed outside of researchers’ own country or community. The policy applies to researchers who have travelled to a different country to conduct research, research with Indigenous populations or their lands, and research on cultural artefacts. The questionnaire can also be requested at the journal’s discretion for any other submissions, even if these conditions are not met.  Please find more information on the policy and a link to download a blank copy of the questionnaire here: https://journals.plos.org/plosone/s/best-practices-in-research-reporting. Please upload a completed version of your questionnaire as Supporting Information when you resubmit your manuscript.

3. If the reviewer comments include a recommendation to cite specific previously published works, please review and evaluate these publications to determine whether they are relevant and should be cited. There is no requirement to cite these works unless the editor has indicated otherwise.

[Note: HTML markup is below. Please do not edit.]

[NOTE: If reviewer comments were submitted as an attachment file, they will be attached to this email and accessible via the submission site. Please log into your account, locate the manuscript record, and check for the action link "View Attachments". If this link does not appear, there are no attachment files.]

To ensure your figures meet our technical requirements, please review our figure guidelines: https://journals.plos.org/plosone/s/figures

You may also use PLOS’s free figure tool, NAAS, to help you prepare publication quality figures: https://journals.plos.org/plosone/s/figures#loc-tools-for-figure-preparation.

NAAS will assess whether your figures meet our technical requirements by comparing each figure against our figure specifications.

# Author comment: Author response to Decision Letter 1

**DOI:** 10.1371/journal.pone.0341752.r002

23 Apr 2026

Dear the ACADEMIC EDITOR:

Please find below the responses to your edits. Line numbers are in relation to the new Manuscript document.

Abstract; please arrange the abstract with the headings and as stated below:

• Introduction/background: (Objective(s) of the study clearly stated): Heading has been added (line 23) and objectives have been described (lines 25-29).

• Methods: Ensure that you mention qualitative and quantitative analysis, sampling method (purposive?) for qualitative analysis and the number of participants. Heading (line 30) and clarification of the qualitative and quantitative data analysis (line 32 & 35), sampling methods (line 31 & 33). The number of interview participants was already stated but hopefully now clearer (line 33-34)

• Procedures to achieve results: unsure what additional information you are asking for that are not in the methods?

• Results Heading added (line 36)

• Conclusion Heading added (line 41)

Introduction; please ensure that you arrange your facts sequentially as stated below:

• Global state of wildlife extinction: Global mention of wildlife extinction has been removed.

• Services – professional obligations have been moved to lines 64-73

• Major challenges - have been rearranged; unknown wildlife admission numbers, professional obligations, current inhibiting factors, wildlife centre relationships.

• Continental state of wildlife and veterinary services: European wildlife levels and veterinary services are no relevant in this UK based project.

• What is obtainable in the UK with regards to wildlife veterinary services? – This is part of the research question (line 97-100)

• Challenges, research questions and objective of the study as the last statement of the last paragraph – Clarified and is final paragraph Line 93-100

Methods

• What were the semi structured questions? Please, include for clarity. All three interview scripts are available on the data share DOI I have clarified this on line 134

• LINE 125; state the meaning of GDPR before abbreviating – Sentence including GDPR has been removed.

• What type of sampling technique was used in the selection of your study participants? Please, state clearly.

• Survey and interview sampling were both purposive, convenience sampling now stated on line 108 and 127.

• Interview quota aim was 12 with a max of 20 interviews per group, now stated on line 130-132 & 136-137.

• Ensure to rephrase this statement lines 170-172” Each interview was recorded audibly and transcribed by Microsoft Teams software during the interview, with post editing for accuracy and verbatim“. - Rephased on lines 150-152

• State the type of device you used in recording Online using a laptop (line 150)

• The average time for the interviews conducted. Already in the manuscript on line 152.

• The statement on data saturation was not clear, state clearly whether data saturation point was attained during the interviews? Was there a point you felt that there was no new information generated during the interviews? Data saturation was achieved for the veterinary professional group but not for the wildlife centre and academic educator groups. Clarification on lines 136 and 145-147.

Results

Construct a table that indicates codes generated, subthemes and themes that emerged from the thematic analysis for a broader perspective of what was done and clarity. – Table created and in Supportive Material – S1 Table Line 228 & 878

Line 238; ensure that you write veterinarian in full in the translation for clarity – corrected to veterinarian(s) or veterinary in all the quotes

The quantitative analysis augments the findings of the qualitative analysis or may even defer. While I agree with the potency of the study. There should not be discussion in the results. Clearly state your findings on the survey (quantitative analysis) separately. Then, state your findings in the qualitative analysis. Finally in the DISCUSSION section you can compare and contrast your findings. This is essential for clarity. – Results and Discussion have been separated.

Line 286; write the full meaning of BVA. – sentence including BVA has been removed.

Write the full meaning of CPD before abbreviating. – Written in full (continual professional development) at the first mention of CPD in Table 2.

Line 314; detrimental effects on students not “of students”. - corrected

Uni? Please, write university in full. – corrected all “uni” to “university”

Your findings should be the basis of your results. Discussions on the findings and your opinions should be strictly related in the discussion section. – Results and Discussion have been separated

Ensure that you write this abbreviation in full “RSPCA”. – RSPCA has been removed

Line 425; ensure that you remove referencing from results “Mullineaux and Haering et al.” - – Results and Discussion have been separated so references are only in discussion.

Line 437; iSpy? removed

Discussion for this manuscript is basically absent which should not be so. Like I mentioned earlier, the survey results should be clearly stated, findings of the qualitative studies should be clearly stated under the themes and subthemes developed while discussions of the findings should be under discussion. – Results and Discussion have been separated

Furthermore, the last paragraph in the discussion should be the limitations of the study. – Limitations moved to end of discussion line 815-823

I hope these edited are sufficient to your requests. Please let me know if I have misunderstood anything.

I look forward to hearing from you.

Rebecca Lang.

Attachment Submitted filename: Response to Reviewers.docx

# Peer review: Decision Letter 1

**DOI:** 10.1371/journal.pone.0341752.r003

**Authors:** Ayi Vandi Kwaghe

30 Jun 2026

Dear Dr.Lang,

Thank you for submitting your manuscript to PLOS ONE. After careful consideration, we feel that it has merit but does not fully meet PLOS ONE’s publication criteria as it currently stands. Therefore, we invite you to submit a revised version of the manuscript that addresses the points raised during the review process.

==============================

**ACADEMIC EDITOR: Authors should ensure that the reviewers comments are properly attended to.**

==============================

Please submit your revised manuscript by Aug 14 2026 11:59PM. If you will need more time than this to complete your revisions, please reply to this message or contact the journal office at plosone@plos.org. When you're ready to submit your revision, log on to https://www.editorialmanager.com/pone/ and select the 'Submissions Needing Revision' folder to locate your manuscript file.

- A letter that responds to each point raised by the academic editor and reviewer(s). You should upload this letter as a separate file labeled 'Response to Reviewers'.
- A marked-up copy of your manuscript that highlights changes made to the original version. You should upload this as a separate file labeled 'Revised Manuscript with Track Changes'.
- An unmarked version of your revised paper without tracked changes. You should upload this as a separate file labeled 'Manuscript'.

If you would like to make changes to your financial disclosure, please include your updated statement in your cover letter. Guidelines for resubmitting your figure files are available below the reviewer comments at the end of this letter.

If applicable, we recommend that you deposit your laboratory protocols in protocols.io to enhance the reproducibility of your results. Protocols.io assigns your protocol its own identifier (DOI) so that it can be cited independently in the future. For instructions see: https://journals.plos.org/plosone/s/submission-guidelines#loc-laboratory-protocols. Additionally, PLOS ONE offers an option for publishing peer-reviewed Lab Protocol articles, which describe protocols hosted on protocols.io. Read more information on sharing protocols at https://plos.org/protocols?utm_medium=editorial-email&utm_source=authorletters&utm_campaign=protocols.

As the corresponding author, your ORCID iD is verified in the submission system and will appear in the published article. PLOS supports the use of ORCID, and we encourage all coauthors to register for an ORCID iD and use it as well. Please encourage your coauthors to verify their ORCID iD within the submission system before final acceptance, as unverified ORCID iDs will not appear in the published article. *Only*  the individual author can complete the verification step; PLOS staff *cannot*  verify ORCID iDs on behalf of authors.

We look forward to receiving your revised manuscript.

Kind regards,

Ayi Vandi Kwaghe, D.V.M., M.V.Sc., P.G.D.E. Ph.D., MPH, FETP

Academic Editor

PLOS One

Journal Requirements:

If the reviewer comments include a recommendation to cite specific previously published works, please review and evaluate these publications to determine whether they are relevant and should be cited. There is no requirement to cite these works unless the editor has indicated otherwise.

Please review your reference list to ensure that it is complete and correct. If you have cited papers that have been retracted, please include the rationale for doing so in the manuscript text, or remove these references and replace them with relevant current references. Any changes to the reference list should be mentioned in the rebuttal letter that accompanies your revised manuscript. If you need to cite a retracted article, indicate the article’s retracted status in the References list and also include a citation and full reference for the retraction notice.

[Note: HTML markup is below. Please do not edit.]

Reviewers' comments:

Reviewer's Responses to Questions

**Comments to the Author**

Reviewer #1: All comments have been addressed

Reviewer #2: (No Response)

**********

2. Is the manuscript technically sound, and do the data support the conclusions?

Reviewer #1: Yes

Reviewer #2: Yes

**********

3. Has the statistical analysis been performed appropriately and rigorously?

Reviewer #1: N/A

Reviewer #2: No

**********

4. Have the authors made all data underlying the findings in their manuscript fully available?

The PLOS Data policy

Reviewer #1: Yes

Reviewer #2: Yes

**********

5. Is the manuscript presented in an intelligible fashion and written in standard English?

Reviewer #1: Yes

Reviewer #2: Yes

**********

Reviewer #1: Pleasure reviwing you revised script.I appreciate the improve quality and readability. I have no further comments

Reviewer #2: 1. Overall Assessment

Major weaknesses:

• Participant bias adequately acknowledged but not fully addressed – the sample likely overrepresents wildlife-interested professionals.

• Survey analysis is purely descriptive – no statistical testing or inferential analysis, limiting its contribution beyond describing the sample.

• Results and Discussion are combined – while acceptable for qualitative work, this sometimes makes it difficult to distinguish raw findings from interpretation.

• Limited generalizability acknowledged but the small interview sample (especially n=7 for wildlife centres) may not have reached full saturation.

• Some overclaiming – e.g., "will require a system change" (Abstract) is strong language for a qualitative study of 409 + 33 participants.

________________________________________

2. Criteria-Based Review

A. Clarity and language

• Acceptable with minor edits.

• Abstract is clear and well-structured.

• Issues:

o Title is overly long and repetitive – consider shortening (e.g., “Influences on UK veterinary professionals' engagement with wildlife medicine: A qualitative study”).

o Some sentences are overly complex (e.g., lines 87–90, 109–112) – simplify for readability.

o Typographical: “promotors” (line 224) should be “promoters” (consistent with Abstract uses “promoters”).

o Fig 2, 3, 5 – word clouds are visually appealing but less informative than bar charts with percentages.

o Some references missing page numbers or DOIs (e.g., ref 4, 19, 20).

B. Originality and significance

• Originality: Moderate-high – This is the first qualitative UK study to systematically explore both promoters and inhibitors of wildlife treatment across multiple stakeholder perspectives (vets, nurses, wildlife centres, educators).

• Significance: High – Wildlife populations are declining; veterinary professionals are critical first responders. The findings have direct implications for RCVS policy, veterinary education curricula, and practice-level protocols.

• Novel contributions:

o Explicit identification of RCVS as a central (but currently underutilized) influence.

o Species bias mapping (hedgehogs most favored, pigeons/rats most disfavored).

o GRPI model application to wildlife medicine in practice.

C. Methods (PLOS ONE key area)

Criterion Present? Quality

Ethical approval Yes (HERC_2024_120) Excellent

Informed consent Yes (written) Excellent

Survey recruitment strategy Clearly described Good

Interview recruitment Described per group Good

Sample size justification Partial (cited 12/saturation but only 7 wildlife centre interviews) Needs clarification

Data saturation statement Not explicitly stated Should add

Thematic analysis approach Braun & Clarke (2006) – clear Good

Positionality statement Yes (line 182–187) Excellent

Data availability Full transcripts + survey deposited Excellent (exemplary)

Pilot interviews Yes Good

Critical improvements needed:

1. Saturation: The authors state they aimed for “at least twelve interviews per group” (line 146) but only achieved 20 (vets), 7 (wildlife centres), and 6 (educators). This needs justification – e.g., did data saturation occur for vets? Was it not achieved for wildlife centres/educators, and how does this limit findings?

2. Survey analysis: The survey is described as “descriptive rather than statistical” (line 139). However, with n=409, basic inferential tests (chi-square, logistic regression) could have identified associations (e.g., between education level and confidence, or practice type and wildlife protocols). This is a missed opportunity.

3. Interview guide availability: The author’s state templates are available at the Data Share link, but they are not included in the manuscript. Recommend including as Supplementary File S1.

4. Coding reliability: Only one researcher coded the data. While positionality is acknowledged, inter-coder reliability or researcher triangulation would strengthen validity. Consider adding a statement about reflexive practices.

D. Results

Weaknesses:

1. No inferential statistics – Table 2 shows percentages but no tests of association. For example:

o Is wildlife CPD uptake associated with practice type?

o Is awareness of RCVS investigations associated with years since graduation?

o These analyses would strengthen the paper.

2. Results and Discussion combined – While acceptable for qualitative research, it makes it harder to trace the raw data to the interpretation. Consider separating into distinct sections or clearly signposting when interpretation begins.

3. Lack of negative cases – The authors acknowledge participant bias (line 203–207) but do not explore whether any interviewees expressed negative or reluctant views towards wildlife. This would have strengthened the analysis.

4. Quantitative data not fully integrated – The survey and interview data are presented separately; a mixed-methods integration (e.g., joint display) would enhance the narrative.

E. Discussion

Problems:

• Overclaiming – “will require a system change” (Abstract) is too strong for a qualitative study of 409 + 33 participants. Suggest “may benefit from” or “would be supported by.”

• Long-term sustainability not addressed – The recommendations (e.g., protocols, CPD) are plausible but no evidence of feasibility or cost-effectiveness is provided.

• No limitations section – This is a significant omission. PLOS ONE requires authors to discuss limitations. Add a dedicated subsection covering:

o Participant bias (self-selection).

o Small wildlife centre/educator sample.

o Single coder.

o No inferential statistics.

o Potential social desirability bias in interviews.

o Limited generalizability beyond the UK.

F. Ethical standards & data availability

• Ethics: Excellent – HERC approval, written informed consent.

• Data availability: Exemplary – Full survey results, interview scripts, and transcripts deposited at Edinburgh DataShare (DOI: 10.7488/ds/8059). This exceeds PLOS ONE requirements.

Minor suggestion: Clarify that interview transcripts were anonymized before deposition.

G. References

Issues:

• Ref 4 (Barnes) – available as a thesis; should include DOI or URL.

• Ref 19 (Ando et al., 2014) – page numbers missing.

• Ref 20 (Guest et al., 2006) – page numbers missing.

• Some references (e.g., 26, 27, 28) are web-based; include access dates (some have them, some don’t). Standardize.

________________________________________

3. Major Required Revisions (for publication)

1. Add a dedicated Limitations section (see E above for content).

2. Clarify data saturation – explicitly state whether saturation was achieved for each stakeholder group; if not, acknowledge this as a limitation.

3. Consider adding inferential statistics to the survey analysis (at least chi-square tests for key associations) or justify why they are omitted.

4. Separate Results and Discussion or clearly delineate when interpretation begins (e.g., “This suggests that…”).

5. Tone down overclaims – replace “will require” with “would benefit from” or “may require” (Abstract and Conclusions).

6. Add interview guides as Supplementary File S1 (or confirm they are at the DataShare link).

________________________________________

4. Minor Revisions

• Typo: “promotors” → “promoters” (line 224).

• Title: shorten to “Influences on veterinary professionals to perform wildlife medicine in the United Kingdom: A qualitative study” (or similar).

• Fig 2: Bar chart with percentages would be more informative than the current unlabelled ordinal ranking.

• Fig 3 and 5: Word clouds are engaging but should be accompanied by a frequency table in the text.

• Line 87–90: Simplify the long sentence about the Canadian policy.

• Line 492–493: Clarify whether “10%” and “7%” refer to survey respondents or interview mentions.

• Standardize access dates for all web references.

• Check all abbreviations: SOPs (standard operating procedures) defined at line 573, but “CPD” not defined until line 287 – define on first use (line 115 or earlier).

________________________________________

**********

what does this mean?). If published, this will include your full peer review and any attached files.

If you choose “no”, your identity will remain anonymous but your review may still be made public.

**Do you want your identity to be public for this peer review?**  For information about this choice, including consent withdrawal, please see our Privacy Policy

Reviewer #1: **Yes:**  Udoka Okpalauwaekwe

Reviewer #2: **Yes:**  okoli solomon chieloka

**********

[NOTE: If reviewer comments were submitted as an attachment file, they will be attached to this email and accessible via the submission site. Please log into your account, locate the manuscript record, and check for the action link "View Attachments". If this link does not appear, there are no attachment files.]

To ensure your figures meet our technical requirements, please review our figure guidelines: https://journals.plos.org/plosone/s/figures

You may also use PLOS’s free figure tool, NAAS, to help you prepare publication quality figures: https://journals.plos.org/plosone/s/figures#loc-tools-for-figure-preparation.

NAAS will assess whether your figures meet our technical requirements by comparing each figure against our figure specifications.

Attachment Submitted filename: review_PONE-D-26-00221R1.pdf

# Author comment: Author response to Decision Letter 2

**DOI:** 10.1371/journal.pone.0341752.r004

28 Jul 2026

Hello and thank you for your suggestions and comments, please find below my responses to each comment.

Major Revisions

1. Comment: Add a dedicated Limitations section (see E above for content).

o Participant bias (self-selection).- Participant bias adequately acknowledged but not fully addressed – the sample likely over-represents wildlife-interested professionals.

o Small wildlife centre/educator sample.

o Coding reliability: Single coder. While positionality is acknowledged, inter-coder reliability or researcher triangulation would strengthen validity. Consider adding a statement about reflexive practices.

o No inferential statistics.

o Potential social desirability bias in interviews.

o Limited generalizability beyond the UK.

Response: Comment seems to have made in relation to Manuscript PONE-D-26-00221 uploaded on 11/01/2026, not the revised manuscript PONE-D-26-00221R1 updated on 23/04/2026.

A Limitations section was added and I have edited it further on line 831-853.

2. Comment: Saturation: The authors state they aimed for “at least twelve interviews per group” (line 146) but only achieved 20 (vets), 7 (wildlife centres), and 6 (educators). This needs justification – e.g., did data saturation occur for vets? Was it not achieved for wildlife centres/educators, and how does this limit findings?

Clarify data saturation – explicitly state whether saturation was achieved for each stakeholder group; if not, acknowledge this as a limitation.

Response: Data saturation between the three groups of interviews has been hopefully described more clearly - line 150-156.

Also in Limitations - line 818-820.

3. Comment: Survey analysis is purely descriptive – no statistical testing or inferential analysis, limiting its contribution beyond describing the sample.

Consider adding inferential statistics to the survey analysis (at least chi-square tests for key associations) or justify why they are omitted.

Response: As the survey sample size was small and it excludes the representation of university educators and wildlife centres I decided to prioritize the thematic analysis of the interviews to produce a richer, deeper qualitative study, which has not previously been performed or published in the UK.

4. Comment: Quantitative data not fully integrated – The survey and interview data are presented separately; a mixed-methods integration (e.g., joint display) would enhance the narrative.

Response: The quantitative survey data is now mixed with the interview thematic results in the Results section - Line 178 – 542

5. Comment: Results and Discussion are combined – while acceptable for qualitative work, this sometimes makes it difficult to distinguish raw findings from interpretation.

Separate Results and Discussion or clearly delineate when interpretation begins (e.g., “This suggests that…”).

Response: Comment seems to have made in relation to Manuscript PONE-D-26-00221 uploaded on 11/01/2026, not the revised manuscript PONE-D-26-00221R1 updated on 23/04/2026.

The results and discussion were separated in the R1 version. Now Results section - Line 178 – 542, Discussion Section line 544 – 869

6 Comment: Tone down overclaims – replace “will require” with “would benefit from” or “may require” (Abstract and Conclusions).

Response: Changed as suggested on line 40 in the abstract and has been removed from the conclusion.

7. Comment: Add interview guides as Supplementary File S1 (or confirm they are at the DataShare link).

Response: Interview scripts available as a supplement document S2 File on line 138 and at https://doi.org/10.7488/ds/8059.

8. Comment: Lack of negative cases – The authors acknowledge participant bias (line 203–207) but do not explore whether any interviewees expressed negative or reluctant views towards wildlife. This would have strengthened the analysis.

Response: Negative or reluctant views from the interviews were scarce but have been highlighted and included in the manuscript, mainly in the inhibitor results.

[Wildlife] is often something that you've never treated or potentially never handled before and I think it's just quite a daunting thing (VP18). Line 388-389.

“We just get so inundated with birds and fledglings. I have a really negative reaction to it… especially pigeons… the winged rats of the world (VP17). Line 403-405

We don't always have enough time to provide as proficient care [to wildlife] as we would for others (VP17). Line 426-427.

9. Comment: Long-term sustainability not addressed – The recommendations (e.g., protocols, CPD) are plausible but no evidence of feasibility or cost-effectiveness is provided.

Repsonse: Section called “Pathways for wildlife engagement” has been added. Line 837-869

Minor Revisions

Comment: Typo: “promotors” → “promoters”

Response: Updated 4 corrections: line 38, 201, 202, 551.

Comment: Title is overly long and repetitive – consider shortening:

Response: Changed to: “Influences on veterinary professionals to perform wildlife medicine in the United Kingdom. A qualitative multifocal study.” – I will email to change the title on data share link

Comment: Fig 2: Bar chart with percentages would be more informative

Response: Redone with percentages - now Fig 3.

Comment: Fig 3 and 5: Word clouds accompanied by a frequency table in the text.

Response: Word clouds now accompanied by a percentage pie-chart - Renamed Fig 4 and Fig 5

Comment: Line 492–493: Clarify whether “10%” and “7%” refer to survey respondents or interview mentions.

Response: Clarified - see line 396-397

Comment: Clarify that interview transcripts were anonymized before deposition.

Response: Statement add - line 159-160

Comment: references.

Ref 4 (Barnes) – available as a thesis; should include DOI or URL.

Ref 19 (Ando et al., 2014) – page numbers missing.

Ref 20 (Guest et al., 2006) – page numbers missing.

Some references (e.g., 26, 27, 28) are web-based; include access dates (some have them, some don’t). Standardize.

Response:

Reference numbers have changed

Ref 2 (Barnes) – DOI added

Ref 15 (Ando et al., 2014) – only page numbers 1-7 available on online article.

Ref 16 (Guest et al., 2006) – page numbers 59–82 added.

I have doubled checked and all web-based references have accessed dates in the format of [cited 2002 Aug 12] as per the Submission Guidelines.

Comment: Check all abbreviations: SOPs (standard operating procedures) defined at line 573, but “CPD” not defined until line 287 – define on first use (line 115 or earlier).

Response: Abbreviation CPD and SOP first used and defined in Table Two on page 11.

UK first used and defined on line 22.

RCVS first used and defined on line 38.

VPS first used and defined on line 107.

CPC first used and defined on line 511

Yours sincerely.

Attachment Submitted filename: Response to Reviewers 3.docx

# Peer review: Decision Letter 2

**DOI:** 10.1371/journal.pone.0341752.r005

**Authors:** Ayi Vandi Kwaghe

6 Oct 2026

Influences on veterinary professionals to perform wildlife medicine in the United Kingdom. A qualitative multifocal study.

PONE-D-26-00221R2

Dear Dr. Lang,

We’re pleased to inform you that your manuscript has been judged scientifically suitable for publication and will be formally accepted for publication once it meets all outstanding technical requirements.

Within one week, you’ll receive an e-mail detailing the required amendments. When these have been addressed, you’ll receive a formal acceptance letter and your manuscript will be scheduled for publication.

An invoice will be generated when your article is formally accepted. Please note, if your institution has a publishing partnership with PLOS and your article meets the relevant criteria, all or part of your publication costs will be covered. Please make sure your user information is up-to-date by logging into Editorial Manager at Editorial Manager® and clicking the ‘Update My Information' link at the top of the page. For questions related to billing, please contact billing support.

If your institution or institutions have a press office, please notify them about your upcoming paper to help maximize its impact. If they’ll be preparing press materials, please inform our press team as soon as possible -- no later than 48 hours after receiving the formal acceptance. Your manuscript will remain under strict press embargo until 2 pm Eastern Time on the date of publication. For more information, please contact onepress@plos.org.

Kind regards,

Chisoni Mumba

Academic Editor

PLOS One

Additional Editor Comments (optional):

Thank you for carefully addressing the comments raised during the review process. I am satisfied that the manuscript has been adequately revised and is suitable for publication. I therefore recommend acceptance.

However, I would ask the authors to attend to the following minor issues during the production/proofing stage:

Line 224: Correct the typo “promotors” to “promoters.”

Title: Consider shortening the title to: “Influences on veterinary professionals to perform wildlife medicine in the United Kingdom: A qualitative study” or similar wording.

Figure 2: Consider presenting the findings as a bar chart with percentages, which may be more informative and easier to interpret than the current unlabelled ordinal ranking.

Figures 3 and 5: The word clouds are visually engaging; however, they should be accompanied by frequency information, preferably as a table in the text or supplementary material, to improve interpretability.

Lines 87–90: Simplify the long sentence describing the Canadian policy to improve readability.

Lines 492–493: Clarify whether the reported “10%” and “7%” refer to the proportion of survey respondents or the frequency of mentions during interviews.

References: Standardize the format of access dates for all web-based references.

Abbreviations: Check that all abbreviations are defined at first use. For example, SOPs (standard operating procedures) are defined at line 573, while CPD is not defined until line 287. CPD should be defined at its first occurrence (line 115 or earlier).

These are minor editorial and presentation issues and do not affect my recommendation for acceptance. They can be addressed during the production/proofing stage.

I congratulate the authors on the successful revision of the manuscript.

Reviewers' comments:

Reviewer's Responses to Questions

**Comments to the Author**

Reviewer #2: All comments have been addressed

Reviewer #3: All comments have been addressed

**********

2. Is the manuscript technically sound, and do the data support the conclusions?

Reviewer #2: Yes

Reviewer #3: Yes

**********

3. Has the statistical analysis been performed appropriately and rigorously?

Reviewer #2: N/A

Reviewer #3: Yes

**********

4. Have the authors made all data underlying the findings in their manuscript fully available?

The PLOS Data policy

Reviewer #2: Yes

Reviewer #3: Yes

**********

5. Is the manuscript presented in an intelligible fashion and written in standard English?

Reviewer #2: Yes

Reviewer #3: Yes

**********

Reviewer #2: 1. Duplicate Content in Results Section

Location: Lines 189–198 and Lines 248–253

Issue: The Results section contains duplicate information about the survey demographics and interview framework. This is a significant redundancy that should be corrected.

First instance (lines 189–198):

"The online survey was completed by 409 veterinary professionals. The results were summarized in Table 1 and 2 and described in combination with the key informant interviews. The interviews were completed with twenty veterinary professionals, seven wildlife centre personnel and six university educators. Codes, subthemes and themes were developed using thematic analysis (S1 Table). A conceptual framework (Fig 1) illustrates three themes that promote engagement with wildlife treatment: Competence, Motivation and Collaborations. Three themes that inhibits engagement with wildlife: Uncertainty, Lack of resources and Conflicts, and a central influence by the RCVS."

Second instance (lines 248–253):

"Interviews were completed with twenty veterinary professionals, seven wildlife centre personnel and six university educators. Codes, subthemes and themes were developed using thematic analysis (S1 Table). A conceptual framework (Fig 5) illustrates three themes that promote engagement with wildlife treatment. Competence, Motivation and Collaboration. Three themes that inhibit engagement with wildlife. Uncertainty, Lack of resources and Conflict, and a central influence by the RCVS."

Recommendation: Delete the second instance (lines 248–253) entirely. The first instance already introduces the framework, S1 Table, and the six themes. Keeping both is repetitive and unnecessary.

________________________________________

2. Figure Number Inconsistency

Issue: The conceptual framework is referred to by different figure numbers in different places:

Location Figure Reference

Results (first instance, line 194) Fig 1

Results (duplicate instance, line 252) Fig 5

Discussion (line 551) Fig 1

Discussion (line 609) Fig 15

Recommendation: Unify all references to Fig 1 for the conceptual framework. After deleting the duplicate instance, ensure all cross-references point to Fig 1.

Reviewer #3: The manuscript is well written, addresses an important one health problem and has relevant findings. However the key limitation of the study is its use of purposive convenience sampling, which may introduce selection bias and limit the generalisability of the findings. Participants with a particular interest in wildlife medicine may have been more likely to respond, meaning the results may not fully represent the views and experiences of the wider veterinary profession in the United Kingdom.

**********

what does this mean?). If published, this will include your full peer review and any attached files.

If you choose “no”, your identity will remain anonymous but your review may still be made public.

**Do you want your identity to be public for this peer review?**  For information about this choice, including consent withdrawal, please see our Privacy Policy

Reviewer #2: **Yes:**  Dr Okoli Solomon.C ,MPH, MSc, FELTP, FCVSN

Reviewer #3: **Yes:**  Sody Munsaka

**********

Attachment Submitted filename: review finalPONE-D-26-00221R2.docx

Attachment Submitted filename: Influences on veterinary professionals.docx

# Editor report: Acceptance letter

**DOI:** 10.1371/journal.pone.0341752.r006

**Authors:** Chisoni Mumba

PONE-D-26-00221R2

PLOS One

Dear Dr. Lang,

I'm pleased to inform you that your manuscript has been deemed suitable for publication in PLOS One. Congratulations! Your manuscript is now being handed over to our production team.

At this stage, our production department will prepare your paper for publication. This includes ensuring the following:

* All references, tables, and figures are properly cited

* All relevant supporting information is included in the manuscript submission,

* There are no issues that prevent the paper from being properly typeset

You will receive further instructions from the production team, including instructions on how to review your proof when it is ready. Please keep in mind that we are working through a large volume of accepted articles, so please give us a few days to review your paper and let you know the next and final steps.

Lastly, if your institution or institutions have a press office, please let them know about your upcoming paper now to help maximize its impact. If they'll be preparing press materials, please inform our press team within the next 48 hours. Your manuscript will remain under strict press embargo until 2 pm Eastern Time on the date of publication. For more information, please contact onepress@plos.org.

You will receive an invoice from PLOS for your publication fee after your manuscript has reached the completed accept phase. If you receive an email requesting payment before acceptance or for any other service, this may be a phishing scheme. Learn how to identify phishing emails and protect your accounts at https://explore.plos.org/phishing.

If we can help with anything else, please email us at customercare@plos.org.

Thank you for submitting your work to PLOS One and supporting open access.

Kind regards,

PLOS One Editorial Office Staff

on behalf of

Dr Chisoni Mumba

Academic Editor

PLOS One
